# Analysis of dynamic changes in retinoid-induced transcription and epigenetic profiles of murine *Hox* clusters in ES cells

**DOI:** 10.1101/gr.184978.114

**Published:** 2015-08

**Authors:** Bony De Kumar, Mark E. Parrish, Brian D. Slaughter, Jay R. Unruh, Madelaine Gogol, Christopher Seidel, Ariel Paulson, Hua Li, Karin Gaudenz, Allison Peak, William McDowell, Brian Fleharty, Youngwook Ahn, Chengqi Lin, Edwin Smith, Ali Shilatifard, Robb Krumlauf

**Affiliations:** 1 Stowers Institute for Medical Research, Kansas City, Missouri 64110, USA;; 2 Department of Anatomy and Cell Biology, Kansas University Medical Center, Kansas City, Kansas 66160, USA

## Abstract

The clustered *Hox* genes, which are highly conserved across metazoans, encode homeodomain-containing transcription factors that provide a blueprint for segmental identity along the body axis. Recent studies have underscored that in addition to encoding *Hox* genes, the homeotic clusters contain key noncoding RNA genes that play a central role in development. In this study, we have taken advantage of genome-wide approaches to provide a detailed analysis of retinoic acid (RA)-induced transcriptional and epigenetic changes within the homeotic clusters of mouse embryonic stem cells. Although there is a general colinear response, our analyses suggest a lack of strict colinearity for several genes in the *HoxA* and *HoxB* clusters. We have identified transcribed novel noncoding RNAs (ncRNAs) and their *cis*-regulatory elements that function in response to RA and demonstrated that the expression of these ncRNAs from both strands represent some of the most rapidly induced transcripts in ES cells. Finally, we have provided dynamic analyses of chromatin modifications for the coding and noncoding genes expressed upon activation and suggest that active transcription can occur in the presence of chromatin modifications and machineries associated with repressed transcription state over the clusters. Overall, our data provide a resource for a better understanding of the dynamic nature of the coding and noncoding transcripts and their associated chromatin marks in the regulation of homeotic gene transcription during development.

The clustered *Hox* genes encode homeodomain-containing transcription factors that confer segmental identity along the primary body axis of both vertebrates and invertebrates (McGinnis and Krumlauf 1992; Gross and McGinnis 1996). They are highly conserved and functionally implicated in mechanisms controlling the regionalization of the body plan of all bilaterally symmetrical animals (de Rosa et al. 1999). A unique feature of clustered *Hox* genes is the direct relationship between their chromosomal organization, expression, and function in time and space during development, termed colinearity (Lewis 1978; Duboule and Dollé 1989; Graham et al. 1989; Simeone et al. 1990; Kmita and Duboule 2003). These nested and ordered domains of vertebrate *Hox* gene expression are coupled to segmentation along the body axis and established during embryogenesis through combinatorial inputs from multiple signaling pathways (Bel-Vialar et al. 2002; Diez del Corral and Storey 2004; Deschamps and van Nes 2005; Wellik 2009; Young et al. 2009; Mallo et al. 2010; Rhinn and Dollé 2012). Conserved axial patterning signals may play a similar role in controlling colinear *Hox* expression in chordates (Wada et al. 1999; Manzanares et al. 2000; Lowe et al. 2003; Ikuta et al. 2004; Seo et al. 2004; Pani et al. 2012).

Insight into mechanisms establishing domains of *Hox* expression arises from in vivo analyses of the response of *Hox* genes to growth factors (Fibroblast Growth Factors [FGFs]) and inducing signals (retinoic acid [RA]) (Conlon and Rossant 1992; Marshall et al. 1992; Isaacs et al. 1998; Pownall et al. 1998; Bel-Vialar et al. 2002). Studies have underscored a key role for RA signaling in transient induction of the early ordered and nested domains of *Hox* expression in the CNS (Diez del Corral et al. 2003). RA signaling is implicated in early positioning of the anterior boundaries of 3′ *HoxB* genes (paralog groups 1–5) (Marshall et al. 1994; Studer et al. 1998; Bel-Vialar et al. 2002; Sirbu et al. 2005) and later in the rostral expansion of the expression domains of 5′ genes in the cluster (Ahn et al. 2014). Direct input of retinoids on transcriptional activity can be mediated through binding of heterodimeric complexes of retinoid (RAR) and retinoid X (RXR) receptors to retinoic acid response elements (RAREs) (Chambon 1994). These RAREs generally have a short direct repeat sequence motif with a variable spacer of two (DR2) to five (DR5) nucleotides. RAREs recruit coactivators (EP300 and CREBBP), corepressors (NCOR1 and NCOR2), and other protein complexes that have inputs into regulation of epigenetic states and modifiers of chromatin accessibility (Kininis and Kraus 2008; Evans and Mangelsdorf 2014).

*Cis*-regulatory analyses have found that the response of *Hox* genes to RA is mediated in part through the presence of RAREs within *Hox* clusters (Alexander et al. 2009; Tümpel et al. 2009). Functional RAREs have been identified adjacent to mammalian *Hoxd4* (Moroni et al. 1993), *Hoxb4* (Gould et al. 1998), *Hoxa4* (Packer et al. 1998), *Hoxb5* (Sharpe et al. 1998; Oosterveen et al. 2003), *Hoxa1* (Langston and Gudas 1992; Dupé et al. 1997), and *Hoxb1* (Marshall et al. 1994; Studer et al. 1994, 1998; Ogura and Evans 1995a,b). In vertebrates, dietary deficiency of retinoids and alterations to enzymes controlling the synthesis and degradation of retinoids display a wide variety of defects associated with changes in patterns of *Hox* expression in the CNS and other tissues (Gale et al. 1999; Niederreither et al. 1999, 2000; Begemann et al. 2001; Grandel et al. 2002; Maden 2002; Linville et al. 2004; Oosterveen et al. 2004; Molotkova et al. 2005; Sirbu et al. 2005; Hernandez et al. 2007; White and Schilling 2008; Rhinn and Dollé 2012). *Hox* genes also regulate components of retinoid signaling (*Aldh1a2*/*Raldh2* and *Rarb*) to set up feedback loops that reinforce positive cross-talk between *Hox* expression and RA signaling (Serpente et al. 2005; Vitobello et al. 2011). In addition to the direct effects of retinoids on *Hox* expression, RA modulates the expression domains of the *Cdx* transcription factors, and these in turn bind to *cis*-elements in the *Hox* clusters to regulate axial expression (Houle et al. 2000, 2003; Lohnes 2003; Young et al. 2009; van de Ven et al. 2011; van Rooijen et al. 2012).

Several studies have demonstrated that teratocarcinoma and embryonic stem (ES) cells can be induced to differentiate upon RA treatment. During this differentiation process, there appears to be a colinear activation of *Hox* genes, such that the 3′ *Hox* genes are sequentially activated before 5′ members of the clusters (Simeone et al. 1990, 1991; Papalopulu et al. 1991). This response reflects the underlying signaling mechanisms related to how axial domains of *Hox* expression are established through the dynamic action of opposing signaling centers during elongation of the vertebrate body axis (Diez del Corral and Storey 2004; Deschamps and van Nes 2005; Young et al. 2009). Hence, understanding the *Hox* response to RA in ES cells is highly relevant for understanding how retinoid signals contribute to the ordered domains of *Hox* expression in neural development (Marshall et al. 1994; Itasaki et al. 1996; Gould et al. 1998; Studer et al. 1998; Gavalas and Krumlauf 2000; Gavalas 2002; Serpente et al. 2005).

A variety of protocols are available to differentiate ES cells using signaling molecules, such as RA, FGFs, activin, and R-spondin (Fraichard et al. 1995; Drab et al. 1997; Wobus et al. 1997; Gottlieb and Huettner 1999; Glaser and Brustle 2005; Kawaguchi et al. 2005). Several previous studies of mouse and human ES cells have characterized gene expression and epigenetic events both in *Hox* clusters and on a genome-wide basis (Chambeyron and Bickmore 2004; Boyer et al. 2005, 2006; Bernstein et al. 2006; Kashyap et al. 2011; Mahony et al. 2011; Gaertner et al. 2012; Mazzoni et al. 2013; Sheikh et al. 2014). The majority of these have focused on events between 24 and 96 h following RA treatment, using doses of 1–10 µM. However, RA concentrations in developing mouse embryos normally range from 16 to 35 nM (Scott et al. 1994; Horton and Maden 1995), and a recent ES cell study has shown that treatments with lower physiological levels of RA more accurately mimic in vivo regulatory events during the early development (Sheikh et al. 2014). In this study, we used 33 nM 9-*cis* retinoic acid in combination with short interval time points in the initial stages of RA-induced differentiation (0–24 h) to characterize the early transcriptional and epigenetic events associated with expression of both coding and ncRNAs in and around the *Hox* clusters. We find that ncRNAs from both strands of *Hox* clusters represent some of the most rapidly induced transcripts in ES cells, and they are associated with dynamic epigenetic changes. At least three of these noncoding regions appear to be directly regulated by RA, and we show coregulation of a ncRNA (*Hobbit1*) and *Hox* coding genes in the *HoxB* cluster is mediated by a shared RARE-dependent enhancer.

## Results

### RA-induced differentiation of murine ES cells

**Figure 1.**
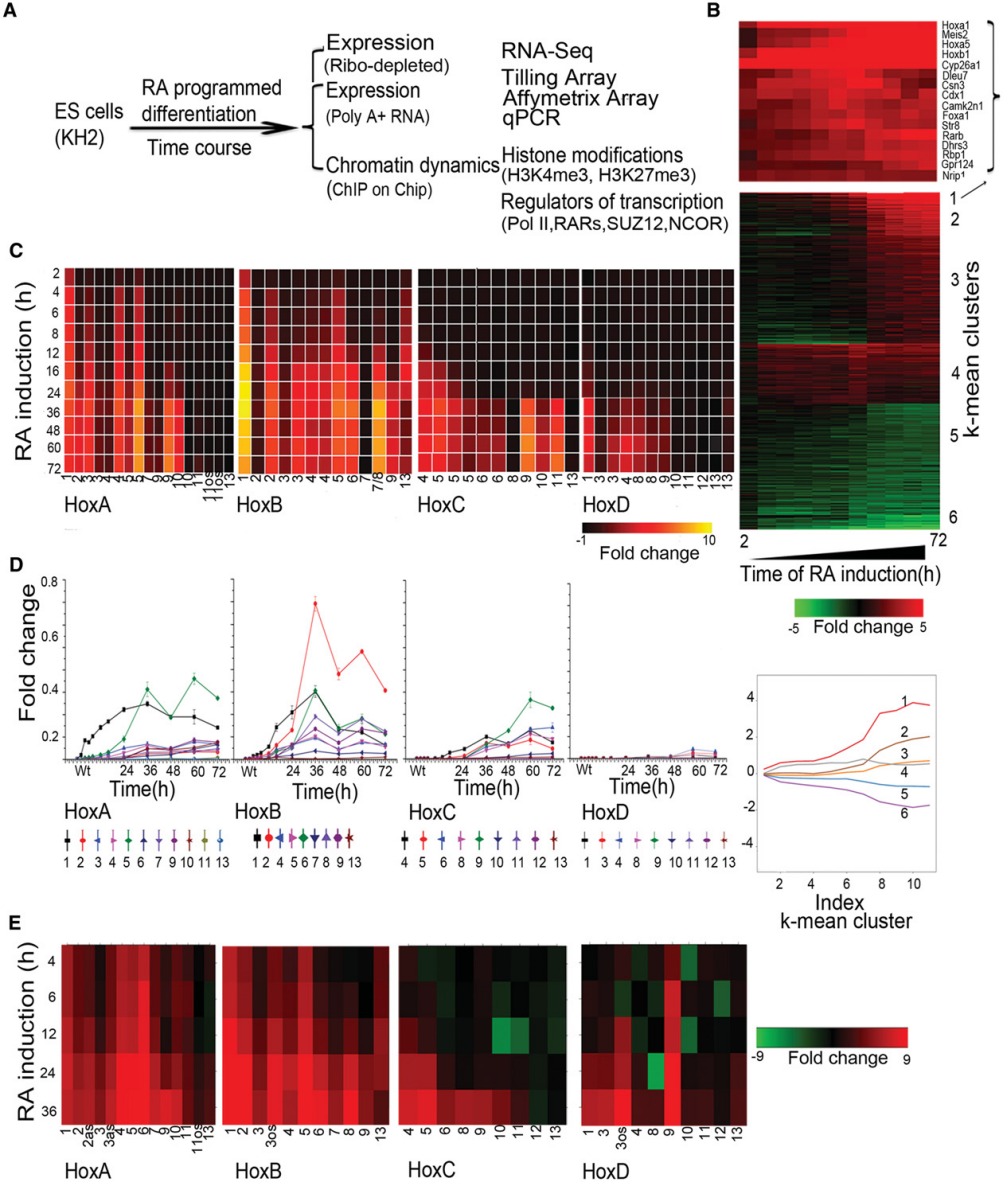
Analysis of changes in *Hox* and global gene expression during RA-induced differentiation of mouse ES cells. (*A*) Flow chart outlining overall experimental strategy. (*B*) Heatmap showing global changes in gene expression upon RA induction compared with uninduced ES cells as analyzed on Affymetrix Mouse Genome 430 2.0 arrays. The *lower* panel shows the distinct induction profiles of six clusters, identified by *k*-means clustering. Expression values are the average value from three independent biological replicates. The *middle* panel displays a heatmap of global changes in gene expression upon RA-induced differentiation; *k*-means clusters are indicated on the *right*. Only clusters with an absolute value of cluster mean >0.5 are shown. The *upper* panel shows changes in the expression profile for 15 of the most rapidly induced genes in cluster 1, which includes *Hox* genes and genes for the cofactors (*Meis*). (*C*) Heatmap of relative changes in *Hox* gene expression upon RA induction compared with uninduced ES cells as analyzed on Affymetrix Mouse Genome 430 2.0 arrays. Genes in the *HoxA* and *HoxB* clusters display a more rapid and robust induction than those of the *HoxC* and *HoxD* clusters. Several genes have multiple probes, and results are shown for each. (*D*) Temporal changes in *Hox* gene expression induced by RA quantitated by TLDA qPCR microfluidics cards. All data points are the average of three biological and two technical replicates. The *y*-axis in all clusters is shown on the same scale and illustrates relative levels of induction between *Hox* clusters. (*E*) RNA-seq analysis of *Hox* gene expression in RA treated ES cells compared to uninduced cells. Fold changes are shown as a heatmap.

We used the programmed differentiation of mouse ES cells to neural fates with RA as a model to investigate the dynamic early expression and epigenetic regulation of *Hox* clusters. A detailed time course using mouse KH2 ES cells (Beard et al. 2006) was performed to examine the transcriptional activity of coding and noncoding regions in and around the four murine *Hox* clusters (Fig. 1A). Samples were harvested between 0 and 72 h after exposure to 33 nM of 9-*cis*-RA. Affymetrix Mouse Genome 430 2.0 arrays were used to monitor global changes in expression profiles, and selected samples were also analyzed by RNA-seq. Applied Biosystems TLDA (Taqman Low Density Array) cards (qPCR), containing probes for the 39 murine *Hox* genes and five endogenous controls, were used to quantitate changes. To monitor transcription from coding and noncoding regions, we designed custom tiling arrays (Agilent) with probes covering both strands of DNA. These spanned the four *Hox* clusters and large areas of their flanking DNA up to the adjacent non-*Hox* coding genes on the 5′ and 3′ sides of each cluster. Applying multiple approaches provided an ability to systematically characterize and validate the qualitative and quantitative changes in the transcriptional profiles of *Hox* clusters induced by RA during early differentiation of ES cells.

We first evaluated the differentiation process based on global changes in gene expression (Supplemental Table S1). Affymetrix data were analyzed for genes showing twofold or more changes in expression levels compared to untreated ES cells using hierarchical clustering and displayed as a heatmap (Fig. 1B). Six clusters, identified by *k*-means clustering, show maximum mean changes in gene expression with distinct induction profiles (Supplemental Fig. S1A,B). Gene Ontology (GO) term analyses of expression profiles indicate that RA progressively drives cells toward a neuro-ectodermal fate (Supplemental Fig. S1C). This is reflected by the up-regulation of a large number of genes related to developmental processes and neurogenesis in concert with the down-regulation of genes related to stem cell development and maintenance, along with negative regulators of cell differentiation. Cluster 1 contains rapidly induced genes expressed upon RA treatment (e.g., *Cyp26a1*, *Rarb*, *Crabp2*, and *Cdx1*) (Fig. 1B), whose functions are associated with pattern specification processes and commitment of these cells to a neuronal fate (Supplemental Fig. S1). *Hox* genes (e.g., *Hoxb1*, *Hoxa1*, and *Hoxa5*) and genes for their cofactors (e.g., *Meis2*) are highly represented in this cluster of rapidly induced genes. These data reveal that the RA-induced differentiation of KH2 ES cells shows a general pattern of gene regulation similar in many aspects to early in vivo phases of the neural development process.

### Monitoring the cellular response to RA

**Figure 2.**
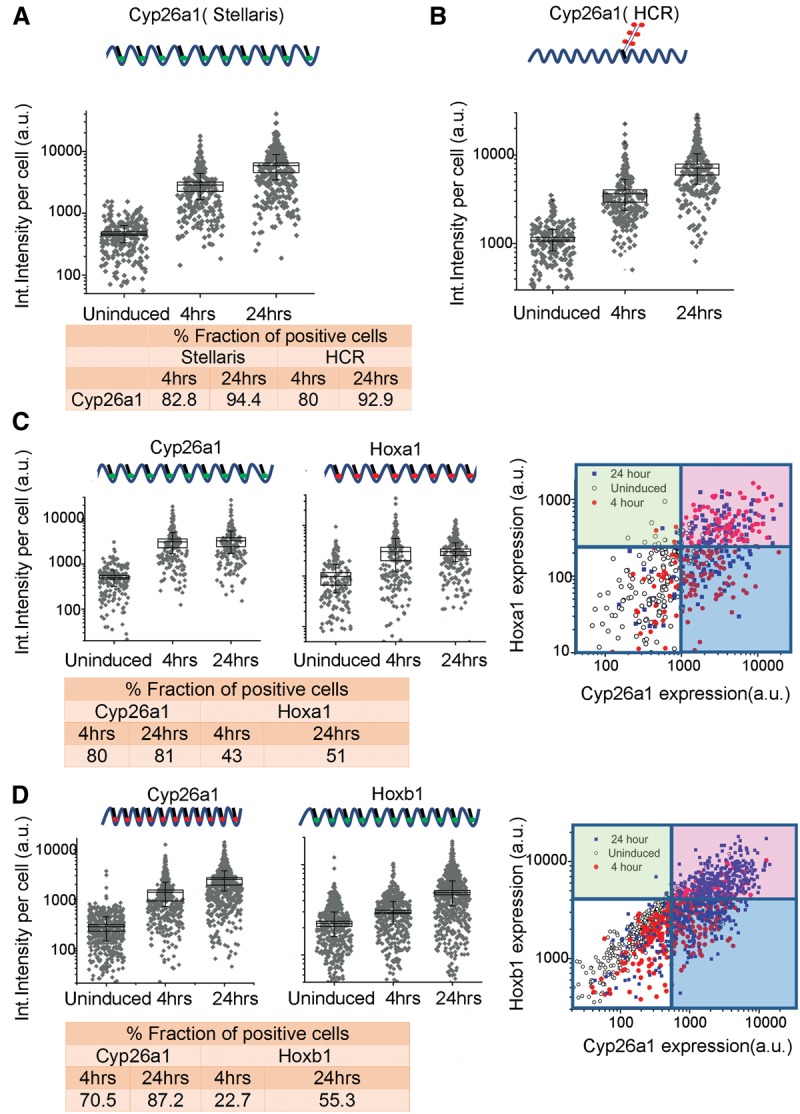
Single-molecule fluorescence in situ hybridization (FISH) analysis of gene expression in ES cells. (*A*,*B*) Quantification of single-color FISH data using either the Stellaris multiprobe approach (*A*) or the hybridization chain reaction (HCR) method (*B*) to measure expression of *Cyp26a1*. Each point represents total integrated intensity in arbitrary units per cell analyzed for uninduced colonies or colonies induced with RA for 4 or 24 h. The *y*-axis is on a log_10_ scale. A 90% threshold of the uninduced distribution is used to estimate levels above which cells are “on” for expression of a gene. (*C*) Dual-color Stellaris FISH analysis using *Cyp26a1-q570* and *Hoxa1-q670* probes. (*D*) Dual-color Stellaris FISH analysis using *Cyp26a1-q670* and *Hoxb1-q570* probes. In *C* and *D*, total integrated intensity in arbitrary units is determined for each color in each cell. Lines on the 2D plot are generated corresponding to the 90% level of the uninduced cells for that color. The white box represents cells that are scored as off for both genes, the red box represents cells on for both genes, and the green and blues boxes represent cells that are positive for a single probe. For *A*–*D*, all experiments used a minimum of 169 total cells from at least nine different colonies for analysis. The fraction of positive cells for each probe and time point are indicated at the *bottom* of the respective panels.

To investigate the level of uniformity of the response of ES cell populations to RA treatment, we used a fluorescence in situ hybridization (FISH) approach for single RNA molecules (Stellaris FISH protocol) developed by Biosearch Technologies (Femino et al. 1998; Raj and Tyagi 2010). This enables us to measure the fraction of cells responding to RA treatment. *Cyp26a1* is the most rapidly induced gene, displaying significant induction within 2 h of RA treatment, and it accumulates high levels of transcripts (Fig. 1B). Therefore, we used it to monitor the efficiency of the RA response. Compared to uninduced ES cells, at 4 h of RA treatment, which is early in the induction process, a majority (83%) of the cells already display a significant *Cyp26a1* signal (Fig. 2; Supplemental Fig. S2). By 24 h, there is a modest additional increase in the fraction of positive cells (<94%) and a major increase in the levels of expression in each cell. We independently validated these results using another single molecule FISH approach, Hybridization Chain Reaction (HCR) (Choi et al. 2010, 2014). HCR analysis with *Cyp26a1* reveals a comparable fraction of positive cells at both 4 h (80%) and 24 h (93%) and similar levels of expression per cell (Fig. 2; Supplemental Fig. S2). These data indicate that the KH2 cells efficiently show a rapid and uniform response to RA, indicating the absence of a major subpopulation of cells resistant to RA or with a delayed response.

*Hoxa1* and *Hoxb1* are also induced early, but they appear after *Cyp26a1* and at much lower levels (Fig. 1B). These lower levels of expression create a challenge for sensitivity of detection by FISH; nonetheless we compared the expression of *Hoxa1*, *Hoxb1*, and *Cyp26a1* at 4 and 24 h time points of RA treatment with uninduced ES cells. In accord with results using the single probe sets, multiplex in situ hybridization with two different Stellaris probe sets (*Cyp26a1/*Hox*a1*; *Cyp26a1/*Hox*b1*) revealed a comparable fraction of cells positive for *Cyp26a1* at 4 h (70%–80%) and 24 h (81%–87%) of RA induction. For *Hoxa1*, 43% of cells scored as positive at 4 h and 51% at 24 h, and levels of expression per cell were similar at the two time points. This correlates with the early induction and rapid plateau in levels of *Hoxa1* observed in the expression profiling and qPCR (Supplemental Fig. S3). For *Hoxb1*, 22% and 55% of cells display expression at 4 and 24 h, respectively, in agreement with the kinetics of the *Hoxb1* induction profile observed by qPCR. Despite the challenges in sensitivity, at 4 h, a significant proportion of the cells display detectable levels of *Hoxa1* and *Hoxb1*, respectively. The lower fraction of *Hoxa1* and *Hoxb1* positive cells at these time points compared to *Cyp26a1* directly correlates with their lower levels of expression and differences in induction kinetics. Correlation plots of the double label experiments show that nearly all of the *Hoxa1* or *Hoxb1* positive cells also display a signal for *Cyp26a1* (Fig. 2), showing they are not separate populations. These results support the idea that the majority of uninduced KH2 ES cells rapidly respond to RA, based on the high fraction of cells positive for *Cyp26a1* at 4 h, and then undergo a progression through the differentiation program, which results in increased levels of expression of *Cyp26a1*, *Hoxa1*, and *Hoxb1* over time. A much larger fraction of the cells are likely positive for *Hoxa1* and *Hoxb1,* but they fall below the level of detection thresholds.

### Temporal dynamics of transcriptional activity in *Hox* complexes

In general agreement with the property of colinearity observed in embryonic development and cellular response to RA (Simeone et al. 1990; Papalopulu et al. 1991; Bel-Vialar et al. 2002; Kmita and Duboule 2003; Alexander et al. 2009), the Affymetrix expression profiling reveals that the order of genes in a *Hox* cluster tends to temporally correlate with their response to RA (Fig. 1C). The detailed time course of RA-induced differentiation allowed us to capture the induction kinetics of genes within each cluster. The *HoxA* and *HoxB* clusters display a more rapid and stronger response to RA than the *HoxC* and *HoxD* clusters, presumably because the *HoxC* and *HoxD* clusters correlate more with posterior genes and later programs of embryogenesis (Fig. 1C). Surprisingly, a strict pattern of temporal colinearity was not followed by some *Hox* genes in the *HoxA* and *HoxB* clusters. For example, expression of *Hoxa5* is observed earlier than that of *Hoxa2* and *Hoxa3*, and *Hoxb5* is detected earlier than *Hoxb3* and *Hoxb4* (Fig. 1C). We independently validated these induction profiles by performing quantitative PCR (qPCR) using Applied Biosystems TLDA cards with *Hox* probes and RNA-seq (Fig. 1D,E; Supplemental Fig. S3). Both assays reveal a similar order of induction, relative levels of gene expression, and variations of strict colinearity, confirming the Affymetrix results. It is worth noting that these observations are based on steady-state levels of mRNA and do not account for possible differences in factors such as RNA stability or transcriptional efficiency of promoters.

### Global transcriptional activity of *Hox* clusters

**Figure 3.**
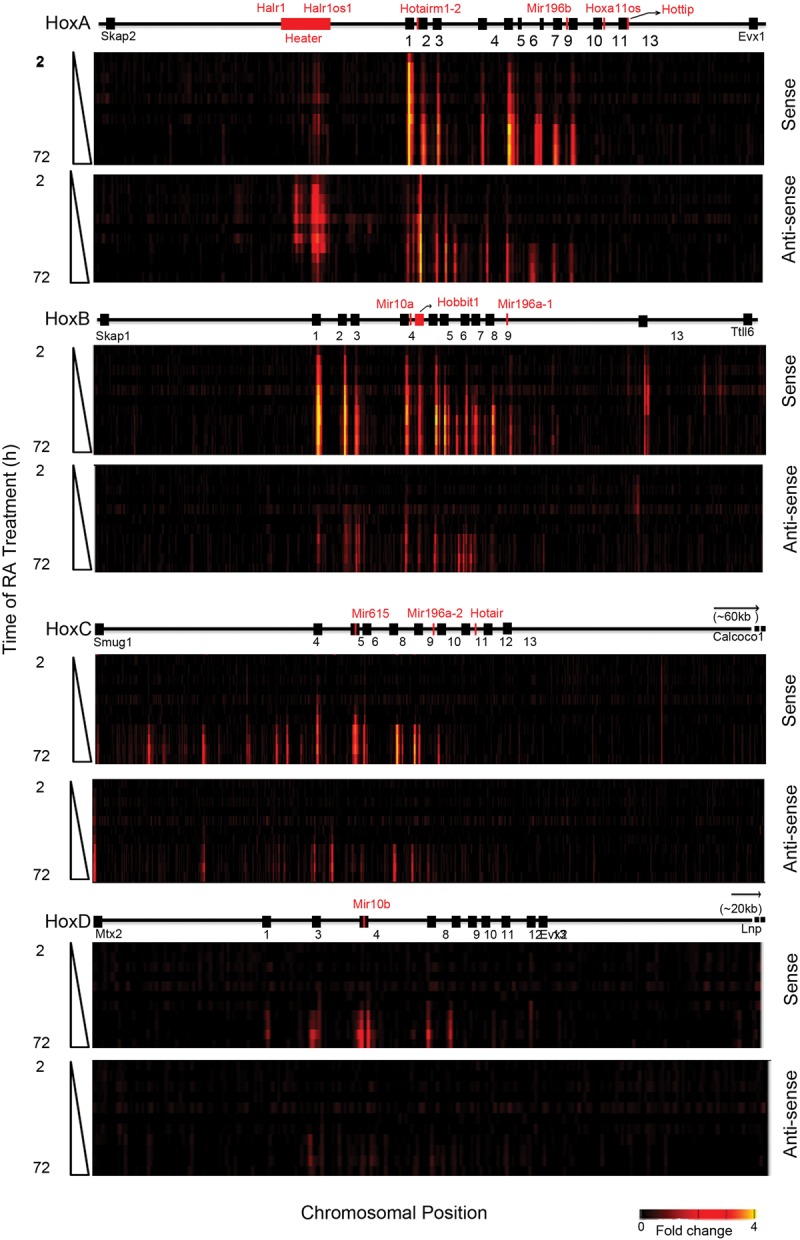
Global transcriptional activity in and around the four *Hox* clusters analyzed by Agilent tiling arrays. Heatmap showing global changes in gene expression over the time course of RA induction compared with uninduced ES cells as analyzed on custom Agilent 2x105K *Hox* tiling arrays. mRNA is labeled with Cy3. Heatmaps are generated on the Integrative Genomics Viewer (IGV) 1.5, where intensity of heat represents relative expression level. *Upper* and *lower* panels in each cluster represent sense and antisense strands, respectively. The *x*-axis denotes the chromosomal position and location of respective *Hox* genes and ncRNAs. The *y*-axis denotes the length of RA treatment in h.

Evidence for lack of strict colinearity in *HoxA* and *HoxB* in response to RA via Affymetrix arrays and qPCR led us to further investigate the nature of the transcriptional profiles spanning the four *Hox* clusters with other methods to avoid concerns about cross hybridization and inability to detail novel transcript isoforms. Therefore, we designed custom tiling arrays that contain probes covering both strands of DNA spanning the four *Hox* cluster regions and large areas of their flanking DNA, up to the adjacent non-*Hox* coding genes on the 5′ and 3′ sides of the clusters. We utilized RNA from the same samples in the time course of programmed differentiation and compared expression levels with undifferentiated ES cells to calculate the fold levels of induction. A heatmap indicating relative levels and chromosomal position of transcripts over the time course of RA treatment reveals a complex and dynamic pattern of transcription (Fig. 3). This detailed temporal analysis allowed us to capture small differences in induction kinetics of genes within each cluster and again revealed the same specific differences in temporal colinearity. The relative order and levels of induction of expression over coding regions correlates very well with the expression patterns obtained by Affymetrix and qPCR analyses (Figs. 1, 3).

The induction profiles indicate a high degree of transcriptional activity from noncoding regions in and around the *Hox* clusters from both sense and antisense strands. Upon induction, extensive intergenic (noncoding) transcription is observed in the *HoxA* and *HoxB* clusters, whereas a lower degree of activity is observed in *HoxC*. The *HoxD* cluster displays no intergenic transcriptional activity at the time points examined (Fig. 3). We observe many previously known noncoding transcripts (*Hotairm1*, *Halr1*, *mir-10a*, *mir-10b*, *mir196a-*1, and *mir196b*) but a large number of novel or unknown transcripts are uncovered in this analysis (Fig. 3; Supplemental Fig. S4). For example, we identified a rapidly induced transcript positioned between *Hoxb4* and *Hoxb5* on the coding strand and named it *Hobbit1* (HoxB4-B5 intergenic transcript1). The expression of several previously characterized ncRNAs, such as *Hottip* and *Hotair*, are not detected. This is likely due to timing, as posterior genes in the *HoxA* and *HoxC* clusters are weakly expressed even after 72 h of RA induction, in accord with their roles in later programs of embryonic development.

There is also induction of a series of noncoding transcripts immediately adjacent to the *HoxA*, *HoxB*, and *HoxC* clusters (Fig. 3). Positioned ∼50 kb 3′ of *Hoxa1*, we observe a large region (∼15 kb) that gives rise to multiple spliced transcripts (at least eight) from both strands of DNA. These transcripts are named *Halr1* (isoforms *1–6*) and *Halr1os1* (isoforms *1* and *2*) (Supplemental Table S7). This includes the three previously identified transcripts, now known as *Halr1* (isoforms *1–3*), found in genome-wide studies of uninduced ES cells (Guttman et al. 2010; Maamar et al. 2013). In uninduced ES cells, there is a low level of transcripts detectable throughout this region, and a rapid increase in transcriptional activity upon RA treatment. We collectively named this region *Heater* (HoxA EArly Transcribed REgion), to designate this dynamic early transcriptional activity near the *HoxA* cluster (Fig. 3, 4A). In the 5′ region flanking *HoxB*, there are a series of weakly expressed early induced transcripts on the sense strand spread throughout the intergenic region between *Hoxb13* and *Ttll6*. The 3′ flanking region between *Hoxc4* and *Smug1* displays a distinct series of intergenic transcripts from the sense strand that appear at later times of induction (after 24–36 h).

**Figure 4.**
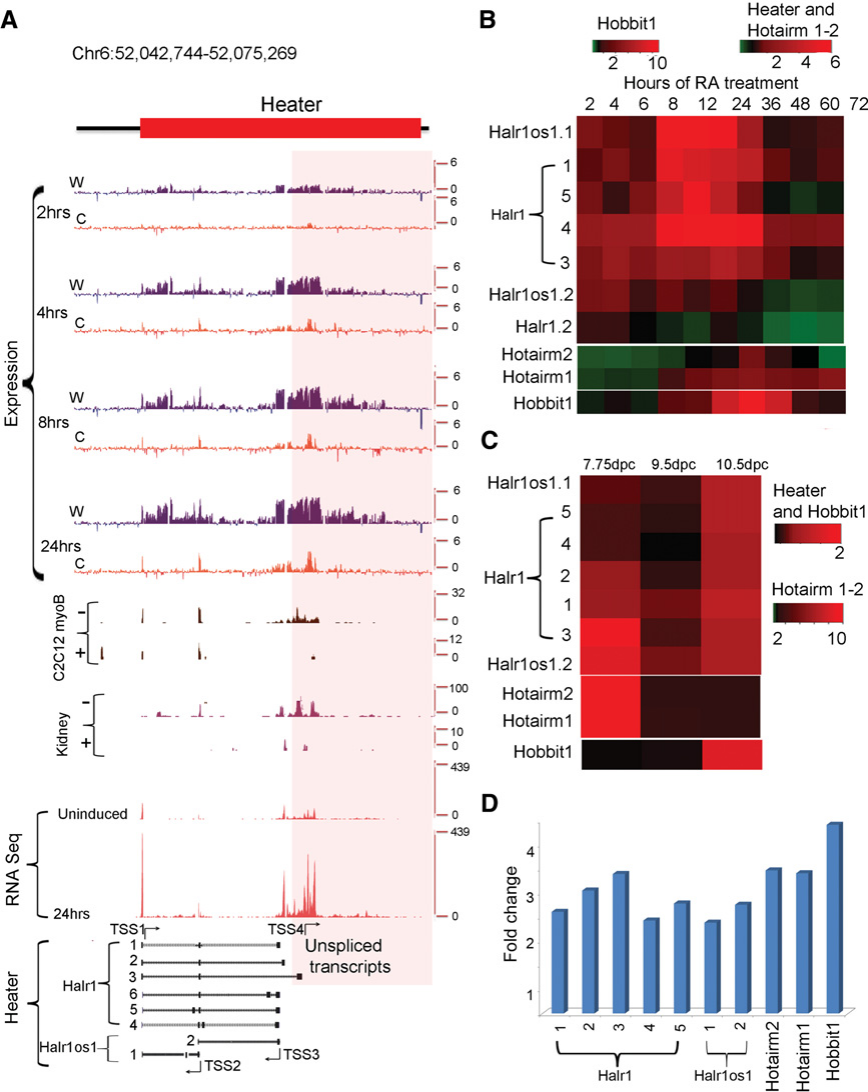
Characterization of *Heater* (HoxA EArly Transcribed REgion) transcripts. (*A*) Transcription from the *Heater* region analyzed by tilling arrays, RNA-seq, and ENCODE. The *top* of the panel shows rapid induction of *Heater* transcripts between 2 and 24 h of RA treatment detected by tiling array profiles. W and C represent Watson and Crick strands, respectively. In the *middle* of the panel is evidence of *Heater* expression in adult kidney and cultured cells based on ENCODE data. + and − indicate opposing strands. RNA-seq data in uninduced and 24-h RA-treated ES cells validate expression profiles observed in tiling arrays. At the *bottom*, multiple *Heater* transcripts, from *Halr1* and *Halr1os1*, are shown schematically with the respective transcription start sites (TSS) and direction of transcription as indicated by arrows. The pink box denotes a region with a large number of unspliced transcripts generated from TSS4. (*B*) Heatmap of qPCR quantitation of *Halr1*, *Halr1os1*, *Hotairm1*, *Hotairm2*, and *Hobbit1* transcripts in RA-induced ES cells. Levels of transcripts are compared against respective transcript levels in uninduced ES cells. *Heater*, *Hotairm1*, and *Hotairm2* are induced at comparable levels, whereas *Hobbit1* shows a higher level of induction in RA-induced differentiation. (*C*) Heatmap of qPCR quantitation of *Halr1*, *Halr1os1*, *Hotairm1*, *Hotairm2*, and *Hobbit1* transcripts in developing mouse embryos. Levels of transcripts are compared against respective transcript levels in 10 dpc embryo. *Heater* and *Hobbit1* are induced at comparable levels, whereas *Hotairm1* and *Hotairm2* show higher levels of induction during mouse embryonic development. Green represents down-regulation; red shows up-regulation. (*D*) Response of *Halr1*, *Halr1os1*, *Hotairm1*, *Hotairm2*, and *Hobbit1* to RA in mouse embryos. The relative response to RA is calculated as fold change in transcript level after treating 10.0 dpc mouse embryos with RA compared to untreated mouse embryos at the same stage.

We further investigated and validated the transcriptional profiles using RNA-seq (Supplemental Fig. S4; Supplemental Tables S2, S3). This allowed us to quantitate the amount of transcripts generated by these sequences. A number of transcripts are detected in uninduced KH2 cells, including *Halr1* (isoforms *1–3*) and *Rps8-ps3* from *HoxA* and *Gm11539* from *HoxB* clusters. *Heater* derived transcripts, *Hotairm1*, *Hotairm2*, *Hoxb3os*, *Hobbit1*, and *Rps8-ps3* are highly induced by RA treatment, and *Gm11539* is slightly down-regulated. The steady state levels of these ncRNAs in both uninduced and RA treated ES cells are significantly higher than their neighboring *Hox* genes (Supplemental Tables S2, S3). This suggests that these ncRNAs are unlikely to reflect run-off transcripts or low levels of background transcription. There are many other ncRNAs on both stands transcribed at a low to moderate level. This highlights an unexpected degree of complexity in the global transcriptional activity in and around *Hox* clusters in addition to the coding regions that needs to be considered in evaluating the regulation of the clustered *Hox* genes.

### Retinoid receptor occupancy in *Hox* clusters

**Figure 5.**
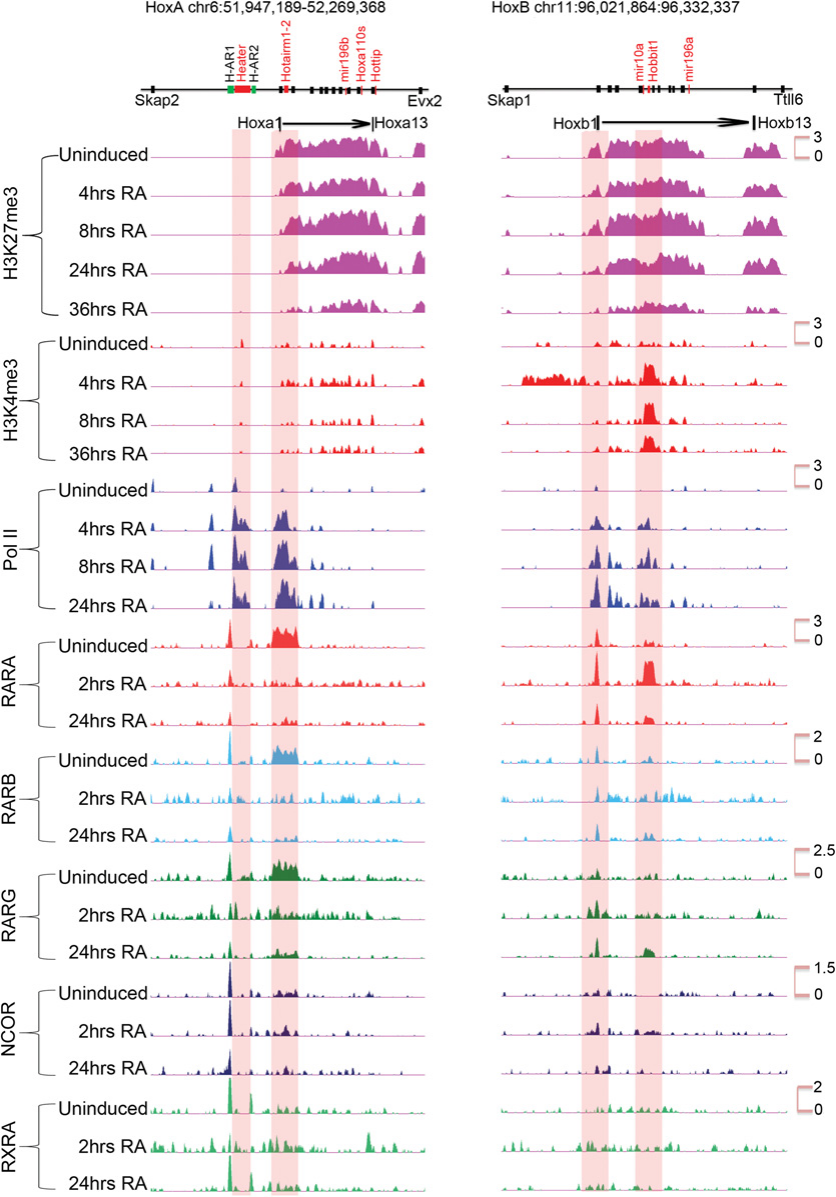
ChIP-on-chip analysis of changes in the epigenetic state and retinoid receptor occupancy of *HoxA* and *HoxB* clusters during RA-induced differentiation. H3K27me3 is used as a repressive mark; H3K4me3 is used as a mark for an active chromatin state, and Pol II is used as a mark of active transcription. Tracks are configured by using windowing function as mean and smoothing windows as 0 pixels in the UCSC Genome Browser. All time points for a given antibody are normalized with the same *y*-axis, and the specific range of each *y*-axis is shown for respective uninduced samples. The schematic at the *top* indicates the relative positions of *Hox* genes, microRNAs, and noncoding transcripts. Within 4 h of RA treatment, there is a rapid gain of H3K4me3 and Pol II, whereas H3K27me3 is gradually lost over 36 h. Many major changes in Pol II occupancy and gain of H3K4me3 are related to *Heater*, *Hotairm1*, and *Hobbit1* noncoding transcripts. Distinct dynamic changes in retinoic acid receptors and the NCOR corepressor is observed over *HoxA* and *HoxB* cluster.

To explore the nature of the transcriptional response to RA, we analyzed dynamic occupancy of retinoid receptors RARA, RARB, RARG, RXRA, and their associated corepressors NCOR1 and 2 on *Hox* clusters. In the *HoxA* cluster, there is a surprisingly large region extending from *Hoxa*1 to *Hoxa3*, including *Hotairm1* and *Hotairm2*, which displays a high level of occupancy of all three RARs but not RXR in undifferentiated cells (Fig. 5; Supplemental Fig. S5). There is also a low level of NCOR binding to this region. There is a dramatic and rapid change in the profile over this region by 2 h, with the broad loss of occupancy of RARA, RARB, RARG, and the recruitment of RXRA (Fig. 5). At 2 and 24 h, RARA, RARB, and RARG occupy other select focused regions in the *HoxA* cluster.

The dynamic patterns of occupancy over the *HoxB* cluster are very different (Fig. 5). There is a well-characterized set of RAREs around *Hoxb1* (Tümpel et al. 2009), and we observe occupancy of the RARs, RXRA, and NCOR over these elements in undifferentiated ES cells. Upon RA treatment, there is very little change in the occupancy profiles in these *Hoxb1* flanking regions, with the exception of expanded recruitment of RARG. In uninduced cells, we also observe a low level of occupancy of RARA, RARB, and RARG in the *Hoxb4–*Hox*b5* intergenic region, which harbors several previously characterized RAREs involved in long-range regulation of multiple *Hox* genes, including *Hoxb4* and *Hoxb5* (Gould et al. 1997, 1998; Sharpe et al. 1998; Oosterveen et al. 2003; Ahn et al. 2014). At 2 h, there is a dramatic gain RARA occupancy (Fig. 5; Supplemental Fig. S6). The RARA occupancy decreased by 24 h of RA treatment and is replaced by increased binding with RARG. This type of RARA-RARG switch has been observed in the differential utilization of retinoid receptors during early versus late stages of embryonic development and ES cell differentiation (Gillespie and Gudas 2007; Kashyap et al. 2011). The rapid recruitment of RARA in the intergenic region between *Hoxb4* and *Hoxb5* correlates with *Hobbit1* transcription and its rapid response to RA (Supplemental Fig. S6). These results reveal distinctly different dynamics of RAR occupancy in the *HoxA* and *HoxB* clusters. Consistent with their weak RA response, the *HoxC* and *HoxD* clusters show very little occupancy in uninduced cells or sustained changes during the first 24 h of differentiation (Supplemental Fig. S8).

### Rapid induction of noncoding transcripts from *Hox* clusters

Many of these ncRNAs display distinct induction kinetics, some of which are as rapid and as strong as the earliest induced *Hox* genes. The *Hobbit1* transcript between *Hoxb4* and *Hoxb5* begins to be induced at 4 h of RA treatment in a manner that parallels *Hoxb4* and *Hoxb5* and is also expressed in the embryo (Fig. 3; Supplemental Fig. S6; Supplemental Tables S2–S4). However, the absolute levels of expression of *Hobbit1* are ∼10-fold higher than *Hoxb4* and *Hoxb5* throughout the differentiation time course.

We found a cluster of three partially overlapping transcripts between *Hoxa1* and *Hoxa2* (Supplemental Fig. S5), one of which corresponds to the previously characterized human *HOTAIRM1* generated from the noncoding strand and involved in myelopoiesis (Zhang et al. 2009). We identified a new splice-variant for *Hotairm1* and a novel transcript (*Hotairm2*), which partially overlaps with *HOTAIRM1* and *Hoxa1*. The tiling arrays, qPCR and RNA-seq reveal that *Hotairm1* and *Hotairm2* transcripts are some of the most rapidly induced RNAs, showing a significant induction by 2 h, similar to *Hoxa1* (Fig. 3; Supplemental Fig. S5C; Supplemental Tables S3, S4). These *Hotairm1* and *Hotairm2* transcripts are expressed in early mouse embryos (7.75 dpc) and decrease to low levels by 9.5 dpc, resembling the kinetics of endogenous *Hoxa1* expression (Supplemental Fig. S5D; Supplemental Table S3). ENCODE data (Rosenbloom et al. 2013) suggest that these transcripts are present in several adult tissues, such as fat pads, heart, kidneys, and spleen.

The *Heater* region generates a complex series of transcripts from different promoters, many with very rapid induction kinetics, appearing around the same time as *Hoxa1* (Fig. 3). This also makes *Heater* RNAs among the earliest *Hox* cluster associated transcripts induced by retinoids. Using a combination of informatics, primer walking, and 5′ RACE, we cloned and characterized eight distinct spliced and polyadenylated transcripts from *Halr1* and *Halr1os1* (Fig. 4A; Supplemental Table S7). The region in *Heater* most proximal to *Hoxa1* also generates a high level of unspliced transcripts. The antisense strand of *Heater* is transcribed at a higher level compared to the sense strand. Analysis (qPCR) of the individual *Heater* transcripts (except isoform *6*) reveals that all of them display a modest level of induction between 2 and 6 h, but at later time points, there are large differences in the dynamics of individual transcript profiles (Figs. 3, 4; Supplemental Fig. S4; Supplemental Tables S2–S4). *Heater* transcripts (*Halr1*; isoforms *1–3*) and *Halr1os1* (isoform *2*) are expressed at high levels in uninduced KH2 cells and show a strong inductive response to RA. Individual *Heater* transcripts also display distinct and dynamic temporal expression profiles in developing mouse embryos (Fig. 4C). ENCODE analyses from the *Heater* region suggest transcription from adult tissues.

To explore whether retinoid signaling has roles in the induction of the *Heater*, *Hotairm1*, *Hotairm2*, and *Hobbit1* noncoding transcripts in developing embryos, we investigated their ability to respond to RA in vivo. Mouse embryos (9.25 dpc) exposed to exogenous RA for 8 h by gavage treatments reveal that all transcripts display a significant up-regulation in response to RA (Fig. 4D). Together, these results indicate that the majority of these intergenic and noncoding transcripts have distinct quantitative and qualitative expression profiles in some way modulated by retinoids.

### Regulation of ncRNAs by retinoids

Next, we investigated how RA might regulate these ncRNAs. Three retinoic acid response elements (RAREs) are positioned in and around *Hoxb4* and *Hoxb5* (Supplemental Fig. S6A), and they are involved in regulating the expression of multiple *HoxB* genes, including both *Hoxb4* and *Hoxb5* (Gould et al. 1998; Sharpe et al. 1998; Oosterveen et al. 2003; Ahn et al. 2014). This raised the possibility that the *Hobbit1* transcript is directly induced by RA through one or more of these RAREs. To functionally test this idea, we measured the levels of *Hobbit1* transcripts in response to RA in neural tissue of a mouse embryo carrying a targeted deletion of the *DE-RARE* (Ahn et al. 2014) and found a significant reduction in expression (Supplemental Fig. S6D). This shows that the *DE-RARE* is a shared *cis-*regulatory element required in vivo for regulating the proper levels of *Hobbit1* and *Hoxb4-*Hox*b5* expression in developing mouse embryos. Further supporting this idea, chromatin immune precipitation (ChIP) assays using the tiling arrays indicate that RARA recruitment throughout this intergenic region increases dramatically within 2 h of RA treatment (Supplemental Fig. S6A). In an analogous case, an RARE located 3′ of *Hoxa1* is required for early activation of the gene in neural tissue (Langston and Gudas 1992; Dupé et al. 1997). This RA-dependent enhancer may also be responsible for the rapid activation of the *Hotairm1* and *Hotairm2* transcripts as their TSS are near those of *Hoxa1*.

**Figure 6.**
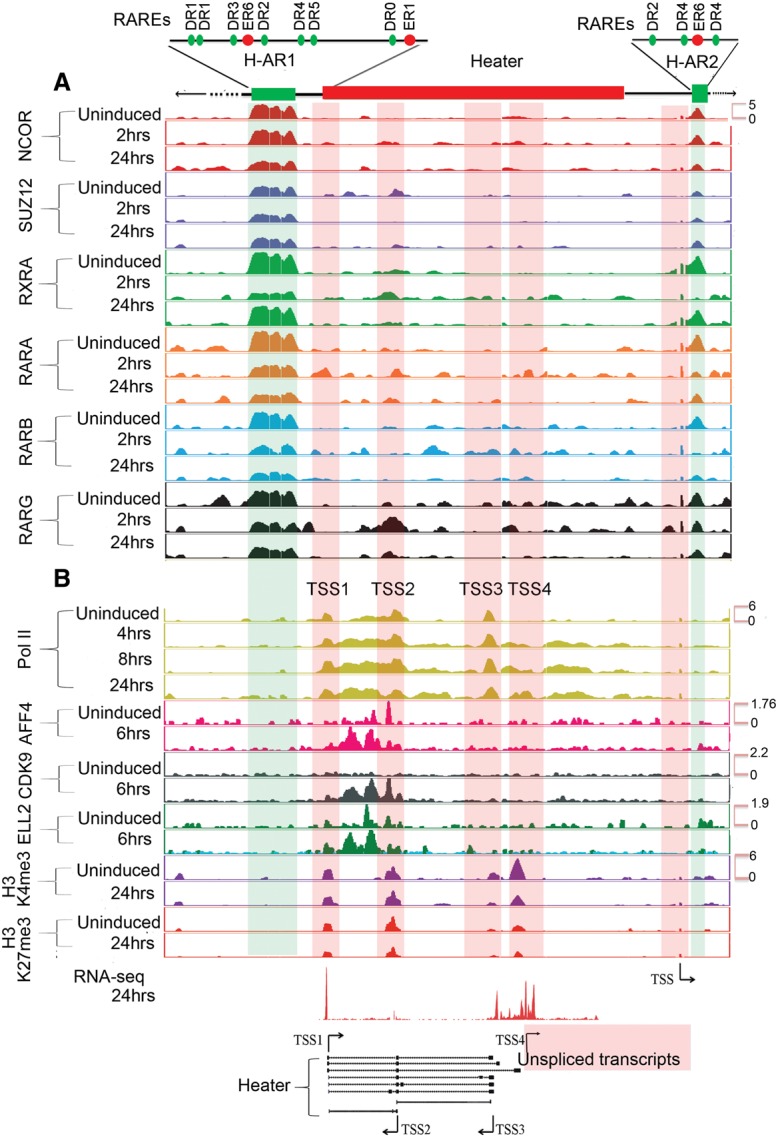
Analysis of changes in the epigenetic state and retinoid receptor occupancy in and around the *Heater* region during RA-induced differentiation. (*A*) Occupancy of retinoic acid receptors (RXRA, RARA, RARB, and RARG) and the NCOR corepressor in and around the *Heater* region. ChIP-on-chip analysis shows a large 2.5-kb region (H-AR1) bound by RAR/RXRs and NCOR upstream of *Heater* and a smaller domain (H-AR2) region downstream from uninduced and RA-treated ES cells. The schematic at the *top* shows the relative positions of these regions to *Heater* and the relative positions of predicted consensus Direct Repeat motifs recognized by retinoid receptors (see Supplemental Fig. S7). (*B*) Dynamic occupancy of Pol II and components of an elongation complex in the *Heater* region. H3K27me3 is used as a repressive mark, H3K4me3 is used as a mark for promoters and active chromatin state, and Pol II is used as a mark of active transcription. Along with Pol II, a bivalent mark formed by H3K4me3 and H3K27me3 is noticeable over TSS1-TSS4. There is a rapid recruitment of the transcription elongation factors (ELL2, AFF4, and CDK9) upon RA induction. Tracks were configured by using a windowing function as mean and smoothing windows as 10 pixels. At the *bottom*, multiple *Heater* transcripts are shown schematically with the respective transcription start sites (TSS) along with RNA-seq at 24 h. The direction of transcription is indicated by arrows. The pink box denotes a region with a large number of unspliced transcripts generated from TSS4. A putative TSS is also apparent in the H-AR2 region.

We investigated potential direct roles for regulation of *Heater* activity by retinoids using ChIP for RARs and NCOR as a potential means to decipher early RA *cis*-regulatory inputs. We observed two distinct RAR/RXR and NCOR binding domains flanking *Heater*, which we refer to as *Heater-Associated Regions* (H-AR). H-AR1 is a large region (2.6 kb) located upstream of TSS1, whereas H-AR2 is positioned downstream from TSS4. Both these domains display a high level of occupancy of RARA, RARB, RARG, RXRA, and NCOR in both uninduced and induced cells (Fig. 6A). In regions flanking these H-ARs are distinct smaller domains that also display evidence for dynamic changes in the occupancy of some of these receptors (Fig. 6A; Supplemental Fig. S7). It is interesting that the occupancy of NCOR shows only modest changes within the first 24 h period. This is consistent with mounting evidence from genome-wide studies that argues against the simple general model whereby the activity of bound RAR/RXR receptors is gated by the occupancy of corepressors, such as NCOR (Mahony et al. 2011; Evans and Mangelsdorf 2014). Therefore, the persistence of NCOR over *Heater* (Fig. 5; Supplemental Fig. S7) and areas of the *Hox* clusters (Fig. 5) is consistent with the idea its occupancy does not necessarily correlate with activity state.

We analyzed the H-ARs for the presence of putative RAREs (Sandelin and Wasserman 2005) and find multiple direct repeat (DR) consensus binding sites spread through the region (Fig. 6A; Supplemental Fig. S7B). There is a highly conserved block of sequence shared with human, rat, orangutan, horse, and dog overlapping the 5′ region of the TSS1 *Heater* transcripts and extending 2.3 kb upstream into the 3′ end of the large RAR/RXR-bound region. Throughout the RAR/RXR-bound region, there are a series of smaller blocks of sequence conservation. These putative RAREs in RAR/RXR-bound regions suggest a direct role in rapid activation of transcripts from the *Heater* region. Intriguingly, a recent study has speculated that *Halr1* (isoforms *1–3*) transcripts from the *Heater* region may be important for potentiating the response of *Hoxa1* to retinoids (Maamar et al. 2013).

### Temporal epigenetic changes in *Hox* clusters

We examined how the transcriptional activity in *Hox* clusters correlated with epigenetic changes in chromatin during RA-induced differentiation. The dynamic nature of the noncoding transcriptional profiles of the *Hox* clusters raises the possibility that they have potential inputs in shaping or responding to epigenetic modifications of chromatin. Toward this goal, we performed ChIP-on-chip assays over a time course of differentiation. Antibodies were used against (1) H3K27me3, a repressive mark; (2) SUZ12, a member of the PRC2 complex and a mark of chromatin silencing; (3) H3K4me3, a mark for active chromatin state; and (4) Pol II, a mark of active transcription.

**Figure 7.**
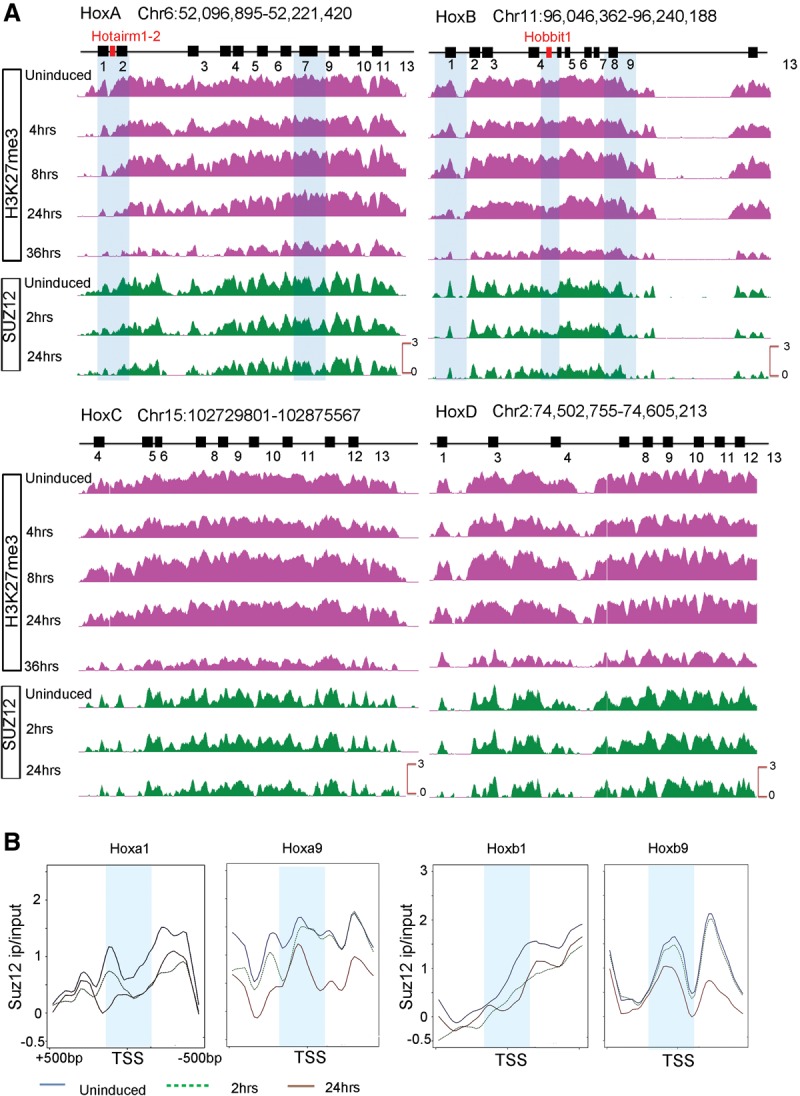
Gradual and progressive loss of repressive marks from the four mouse *Hox* clusters during RA-induced differentiation. (*A*) ChIP-on-chip analysis shows a gradual loss of the H3K27me3 repressive mark and SUZ12 occupancy over each *Hox* cluster upon RA treatment. Although many of the *Hox* genes are expressed early in the time course, over a whole cluster, H3K27me3 is greatly reduced by 36 h of RA treatment. The gradual loss of repressive marks is observed from anterior to posterior genes in a *Hox* cluster over a time course correlating with colinearity. (*B*) Kinetics of reduction of SUZ12 occupancy over TSS of *HoxA* and *HoxB* genes in paralogy groups 1 and 9 during RA-induced differentiation of ES cells. Anterior *Hox* genes rapidly lose SUZ12 over their TSS, as illustrated by changes for *Hoxa1* and *Hoxb1* at 2 h of RA treatment; whereas posterior genes show little change over their TSS in this time frame. The differences in the kinetics of loss of SUZ12 between genes correlates with their respective time of activation. A 500-bp region around the TSS is shown, and a 50-bp region around TSS is marked by a light blue band. The *y*-axis shows relative levels of occupancy of SUZ12.

In accord with published studies (Boyer et al. 2006; Lee et al. 2006; Soshnikova and Duboule 2008; Kashyap et al. 2011), in undifferentiated ES cells, the distribution of the H3K27me3 and SUZ12 repressive marks are widely spread over all four *Hox* complexes (Figs. 5, 7; Supplemental Fig. S8). These marks of a repressed state appear to be confined to the regions spanning the coding genes and are not observed in 3′ or 5′ flanking regions immediately adjacent to the clusters. The one exception is a peak of SUZ12 occupancy over H-AR1 (Figs. 5, 6). This is consistent with the need to maintain *Hox* genes in a silent or inactive state to prevent them from inducing differentiation of these pluripotent cells.

Over the initial 24 h period of RA treatment, there is a progressive decrease in the levels of H3K27me3 over the most 3′ genes, *Hoxa1* and *Hoxb1* (Figs. 5, 7). However, surprisingly, this epigenetic mark remains over many of the genes in the *HoxA* and *HoxB* clusters (Figs. 5, 7), despite the fact that nearly all of the genes are expressed at 24 h (Figs. 1, 3). This repressive mark is slowly erased with longer RA treatments and nearly gone by 36 h (Figs. 5, 7). A similar temporal pattern for the removal of H3K27me3 is also observed for the *HoxC* and *HoxD* clusters (Supplemental Fig. S8). Consistent with the results for *Hox* clusters, we also noted a similar delayed removal of this H3K27me3 mark over the rapidly induced and highly expressed *Cyp26a1* gene (Supplemental Fig. S9), indicating that this observation is not restricted to *Hox* clusters.

We expected the H3K27me3 mark would reflect progressive changes in PcG occupancy that tightly correlated with activation of *Hox* expression. This led us to look at the occupancy of SUZ12, a component of PRC2, as an alternative means of monitoring PcG-mediated repression and its correlation with gene activity. Focusing on the transcription start sites (TSS) of the *Hox* genes, we observe a reduction in the SUZ12 occupancy in the vicinity of the TSS at 2 h of RA treatment for the rapidly induced 3′ *Hox* genes, such as *Hoxa1* and *Hoxb1* (Fig. 7B). Genes in the middle of the cluster, paralogous groups 3–5, begin to display changes in SUZ12 occupancy at 2 h that correlates with their induction profiles (Supplemental Fig. S9). The more posterior genes, such as *Hoxa9* and *Hoxb9*, do not show a reduction at 2 h, but display a marked change in occupancy by 24 h (Fig. 7). Similarly, changes in the occupancy of SUZ12 on the TSS of *Cyp26a1*, *Hobbit1*, and *Hotairm1* also correlate with their induction profiles (Supplemental Figs. S9, S10). Hence, in contrast to the H3K27me3 data, we found a strong correlation between the progressive and rapid removal of SUZ12 over the promoters of all of the *Hox* genes, ncRNAs (*Hobbit1*, *Hotairm1*), and *Cyp26a1* and the timing of their activation (Fig. 7; Supplemental Figs. S9, S10). This implies that in this ES cell context, occupancy of SUZ12 is a better indicator of progressive changes in PcG-mediated repression than the H3K27me3 mark, which takes much longer to be removed. This further suggests that in general, the progressive removal of repressive marks, such as SUZ12 and H3K27me3, occurs first over TSS regions, whereas removal from the whole cluster is slower compared to the rate of gene activation.

With respect to active marks, there is evidence for the occupancy of Pol II over some genes in the *HoxA*, *B*, and *D* clusters in undifferentiated cells (Fig. 5; Supplemental Fig. S8). The polymerase is concentrated near the TSS and not located over the entire gene. This pattern is characteristic of paused Pol II, suggesting that polymerase has initiated but is waiting for signals to potentiate elongation. There is also a low level of H3K4me3 over the regions, consistent with the idea that this mark in mammalian cells frequently correlates with Pol II initiation at the promoter. In the case of *Hoxa1*, we have shown the gene is rapidly induced by RA through the recruitment of the Super Elongation Complex to the promoter, which stimulates elongation of the paused polymerase (Lin et al. 2011). In addition, we demonstrated that many of the most rapidly RA-induced genes in the ES cells are similarly regulated by transcriptional elongation and not by initiation (Lin et al. 2011). The co-occupancy of paused Pol II and H3K4me3 has predictive value for future gene expression in ES cells and *Drosophila* (Gaertner et al. 2012). Hence, the presence of Pol II and H3K4me3 over *Hox* genes, such as *Hoxa3*, *Hoxa5*, and *Hoxa13* suggests that they too may be induced by regulation of elongation of paused polymerases.

During the initial 24 h of differentiation, there is a progressive increase in occupancy of Pol II over genes. Consistent with the general colinear activation of *Hox* genes, this spreads from anterior to posterior genes in the *HoxA* and *HoxB* clusters upon increased length of RA treatment (Fig. 5). There is a similar progressive increase in H3K4me3. However, there are some unique features in the profiles observed over the *HoxA* and *HoxB* complexes that appear to correlate with ncRNA transcripts. At 4 h of RA treatment, Pol II is spread over a wider chromatin domain than expected if it is only present on the *Hoxa1* and *Hoxa2* gene body and promoter. The intergenic region also includes *Hotairm1* and *Hotairm2*. This suggests that Pol II is active on all of these transcriptional units at this early stage of induction, explaining the broad region of occupancy (Fig. 5; Supplemental Fig. S5). In *HoxB*, there is a rapid recruitment of H3K4me3 and Pol II in the intergenic region between *Hoxb4* and *Hoxb5*, which correlates with *Hobbit1* transcription (Fig. 5; Supplemental Fig. S6).

The *HoxC* cluster shows little or no evidence of Pol II occupancy or change of H3K4me3 marks over this time course (Supplemental Fig. S8), which is consistent with the slow or delayed response of genes in this cluster upon RA treatment (Fig. 1). In the *HoxD* cluster near TSS, we detect more bivalent marks over genes (H3K4me3 and H3K27me3). This may indicate that the *HoxD* cluster is ready for activation in later stages. Together, this analysis illustrates the diversity in epigenetic states between each of the *Hox* clusters and reveals that activation of ncRNAs has an impact in the epigenetic regulation of the *Hox* clusters.

### *Heater* and epigenetic changes

Examining epigenetic changes flanking the *Hox* clusters, we focused on *Heater*. In *Heater*, there are four regions showing both H3K4me3 and H3K27me3 marks (bivalent state) and Pol II occupancy that correlate with the TSSs associated with transcription units in *Heater* (Fig. 6B). In uninduced cells, there is evidence for paused Pol II over the *Heater* transcribed region, indicating that some of these transcripts may also be regulated by transcriptional elongation in response to RA (Fig. 6). RA induces a rapid increase in occupancy of Pol II spread over the *Heater* region. Consistent with low levels of *Heater* transcription in ES cells, we detect occupancy of the elongation factors, AFF4 and ELL2, over the TSS2 region. Upon RA treatment, these factors along with CDK9, another elongation factor, increase their level of occupancy over the TSS2 transcription unit and are also rapidly recruited to TSS1 (Fig. 6B). This may underlie the rapid and synchronous response of *Heater* transcripts to RA, as shown for paused polymerase in other contexts (Chopra et al. 2009; Levine 2011; Lin et al. 2011; Lagha et al. 2013). The H-AR2 region flanking *Heater* also displays Pol II occupancy and bivalent H3K4me3 and H3K27me3 marks (Fig. 6). Upon RA treatment, there are dynamic changes in AFF4 and ELL2 occupancy. Hence, the H-AR2 region may also contribute to regulation of the *Heater* response to RA.

Together, these data further highlight the observation that the dynamics of binding of transcription factors and changes in epigenetic marks also correlate with expression of noncoding transcripts. This suggests they play roles in regulating noncoding regions within the *Hox* clusters in addition to the coding transcripts.

## Discussion

In this study, we have analyzed the dynamic patterns of transcription in and around the four *Hox* clusters during the early stages of retinoid-induced differentiation of mouse ES cells into neuronal fates. Single RNA molecule fluorescence in situ hybridization approaches using probes for *Cyp26a1* show that the majority of individual KH2 ES cells respond rapidly to RA in a relatively synchronous manner. The detailed time course revealed novel transcriptional and epigenetic changes. Among these findings, expression of ncRNAs in ES cells represents some of the most rapidly induced transcripts by RA and are associated with dynamic epigenetic changes observed over the *Hox* clusters. These noncoding transcripts are also expressed in mouse embryos and respond in vivo to RA treatment. Our data, showing the presence of adjacent RAREs and retinoid receptor occupancy, suggest that the *Heater*, *Hobbit1*, *Hotairm1*, and *Hotairm2* noncoding regions are directly regulated by RA. In support of this idea, deletion of the *DE-RARE* in the endogenous *HoxB* locus alters the expression and RA response of *Hobbit1*. These results on the dynamic nature of noncoding transcription in the *Hox* clusters raise the possibility they may have roles in shaping or potentiating epigenetic modification in these regions. This has important implications for understanding the complex and dynamic regulation of *Hox* clusters in differentiation and development.

With respect to *Hox* coding regions, in general, there is a colinear order in the activation of gene expression during RA-induced differentiation. However, surprisingly, our analyses uncovered several cases in which genes display a lack of strict temporal colinearity based on steady-state levels of expression. This is clearly observed for the Group 5 genes in the *HoxA* and *HoxB* clusters, which are expressed earlier than their respective Group 3 genes (Figs. 1, 3). This disparity in timing does not account for possible differences in parameters, such as RNA stability or transcriptional efficiency of promoters. Epigenetic changes are associated with noncolinear gene activation and include rapid loss of SUZ12 over the TSS and gain of H3K4me3 and elongating Pol II. Hence, it will be interesting to explore the mechanisms associated with these apparent alterations in colinearity.

The analyses of *Hox* gene promoter regions during RA-induced differentiation reveals that diverse mechanisms appear to function in potentiating their response. Some of the fastest responding genes, *Hoxa1* and *Hoxa5*, are induced by modulating the elongation of paused polymerase, whereas others, *Hoxb1*, *Hoxb4*, and *Hoxb5* display rapid recruitment of Pol II (Fig. 5; Supplemental Fig. S3; Lin et al. 2011). These *Hox* genes displaying a rapid RA response have RAREs associated with their loci. This suggests that induction using paused polymerase and rapid recruitment of Pol II to promoters is achieved in part through integrating direct transcriptional inputs of retinoid signaling.

The mechanisms for regulation by retinoids and other nuclear hormone receptors are themselves diverse and involve the processes of both activation and/or repression to modulate the recruitment and activity of Pol II (Kininis and Kraus 2008; Evans and Mangelsdorf 2014).

Furthermore, in the presence of ligand, there is often a dramatic genome-wide reorganization in receptor occupancy associated with their regulatory roles in cell type-specific gene expression involving loss from preexisting sites, recruitment to new locations, and altered affinities for coactivators and corepressors (Kininis et al. 2009; Mahony et al. 2011; Uhlenhaut et al. 2013). Consistent with this, our analysis has shown that there are dynamic changes in the occupancy profiles of RARs (A, B, and G) over the *HoxA* and *HoxB* clusters. These receptors are bound to the *Hoxa1–*Hox*a3* region in uninduced ES cells and rapidly lost by 2 h of RA treatment (Fig. 5). In contrast, RARA is rapidly recruited to the *Hoxb4–*Hox*b5* intergenic region by 2 h of RA treatment. *Hoxb1* shows constitutive binding of RARA, RARB, and RARG irrespective of RA treatment. This illustrates that multiple mechanisms are implicated in coordinating the temporal regulation of *Hox* genes in response to RA.

Paused polymerase, rapid recruitment of Pol II, and dynamic changes in RAR occupancy also play roles in regulating transcripts from the *Heater*, *Hotairm1*, and *Hobbit1* noncoding regions in a manner similar to the regulation of *Hox* coding regions. There is evidence of paused Pol II over some TSS of *Heater*, as in uninduced cells; there is preloading of Pol II, AFF4, and ELL2 that rapidly increases their occupancy upon RA treatment (Fig. 6). There is also an increase in CDK9 occupancy upon RA treatment, consistent with a role for control of elongation in modulating transcription in this noncoding region. The *Heater Associated Regions* (H-AR1/2) display extensive occupancy of retinoic acid receptors (RXRA, RARA, RARB, and RARG) and contain a number of RARE Direct Repeat motifs (Fig. 6; Supplemental Fig. S7). Hence, the extremely rapid response of *Heater* to RA appears to be mediated by a combination of paused polymerase and direct inputs from retinoid signaling in a manner analogous to *Hoxa1* 50 kb downstream. The *Heater*, *Hotairm1*, *Hotairm2*, and *Hobbit1* regions are all associated RAREs that may be involved in mediating their rapid response. *Hobbit1* is positioned in the intergenic region between *Hoxb4* and *Hoxb5*, flanked by the *DE-* and *B4U*-*RAREs*. Our analysis shows the *DE-RARE* is required for the response of *Hobbit1* to RA in mouse embryos (Supplemental Fig. S6). This illustrates a case in which the regulation of coding (Ahn et al. 2014) and ncRNAs in *Hox* clusters is mediated by a shared *RARE*-dependent enhancer.

We have focused on understanding regulation of these ncRNAs, but it is worth noting that functionally three transcripts (*Halr1*; isoforms *1–3*) located in the *Heater* region have been implicated in regulation of *Hoxa1.* Knockdown of these *Halr1*RNAs leads to increased *Hoxa1* levels in uninduced ES cells, and RA treatment alters this relationship (Maamar et al. 2013). The *Heater* region has a very complex pattern of transcription, as we have characterized at least eight spliced variants generated from three different TSS on both strands. In addition, there are multiple unspliced transcripts induced from a separate TSS (Fig. 4). Therefore, changes in the expression levels and splice variants of different transcripts induced by RA from the *Heater* region may be responsible for uncoupling of *Halr1*expression and *Hoxa1* regulation.

Several previous studies have characterized epigenetic events, both genome-wide and *Hox* cluster-specific, associated with RA-induced differentiation of mouse and human ES cells (Boyer et al. 2005, 2006; Bernstein et al. 2006; Kashyap et al. 2011; Mahony et al. 2011; Gaertner et al. 2012; Mazzoni et al. 2013; Sheikh et al. 2014). Nearly all of these have focused on events after 24 h of RA treatment or use RA in combination with longer (3–8 d) embryoid body differentiation protocols. In this study, we characterized the early dynamic epigenetic events (0–24 h) associated with expression of both coding and ncRNAs in and around the *Hox* clusters. Previous studies have documented the loss of H3K27me3 from *Hox* clusters between 24 and 96 h of differentiation, leading to the assumption that removal of this repressive mark is an early event needed for activation of *Hox* genes.

Indeed, over the initial 24 h period, we observe a progressive decrease in the levels of H3K27me3 over the most 3′ genes, *Hoxa1* and *Hoxb1* (Figs. 5, 7). However, surprisingly the rate of removal of H3K27me3 over all four *Hox* complexes is slow compared with the activation of coding and noncoding transcripts or the appearance of the activation marks (H3K4me3) and Pol II occupancy. The occupancy of the SUZ12, a component of the PRC2 complex, shows a similar slow rate of removal over all four *Hox* clusters. However, examining the TSS regions of *Hox* genes, ncRNAs (*Hobbit1* and *Hotairm1*) and *Cyp26a1*, indicates that there is a temporal correlation in the removal of SUZ12 from TSS and the respective timing of gene expression. Rapid removal occurs first over 3′ genes, such as *Hoxa1* and *Hoxb1*, and progressively is removed from more 5′ genes at later time points (Fig. 7; Supplemental Fig. S10). This indicates that in general, the progressive removal of repressive marks such as SUZ12 and H3K27me3 occurs first over TSS regions, whereas removal from the whole cluster is slower compared to the rate of gene activation. Similar findings with *Cyp26a1* (Supplemental Fig. S9) indicate that this observation is not restricted to *Hox* clusters. This raises the possibility that removal of SUZ12 at promoters might be done through active participation of MLLs and demethylases, whereas over the whole cluster, these marks are removed by patterning signals or passively during transcription and subsequent cell division. Our findings imply that in this ES cell context, occupancy of SUZ12 is a better indicator of progressive changes in Polycomb-mediated repression than the H3K27me3 mark, which takes much longer to be removed.

We find that rapid induction of noncoding transcripts and reorganization of domains of retinoid receptor occupancy impact the epigenetic state of chromatin in *Hox* clusters. For example, the *Hoxb4–*Hox*b5* intergenic region containing *Hobbit1* shows a dramatic gain of H3K4me3 after 4 h of RA treatment along with the recruitment of RARA and Pol II. In the case of *Hotairm1*, there are low levels of H3K27me3 in uninduced ES cells compared to the adjacent *Hoxa1* and *Hoxa2* genes that rapidly declines upon RA treatment (Supplemental Fig. S5A). It is embedded in a region spanning *Hoxa1–*Hox*a3* that displays broad occupancy of retinoid receptors and paused polymerase in uninduced cells. In the *Heater* region, there is paused Pol II and co-occupancy of H3K4me3 and H3K27me3 marks on multiple TSS in uninduced ES cells (Fig. 6). These bivalent states persist upon RA induction. This shows that the bivalent state can be associated with synchronous and rapid induction of noncoding transcripts in differentiating ES cells.

These analyses of early events associated with RA-induced differentiation illustrate the interplay between RARE *cis*-regulatory elements, rapidly induced noncoding transcripts from both strands, and dynamic epigenetics changes in shaping the overall expression profile and epigenetic state in an around *Hox* clusters.

## Methods

### RNA isolation

RNA isolation was performed using TRIzol (Life Technologies) and later purified by the RNeasy Kit (Qiagen). RNA was tested for integrity and concentration using the RNA 6000 Nano Assay and RNA LabChips on the Agilent Bioanalyzer 2100 (Agilent Technologies).

### Affymetrix microarray analysis

Two hundred nanograms total RNA was labeled, and cRNA targets were generated from total RNA samples using the MessageAmp III RNA Amplification Kit (Applied Biosystems/Ambion), according to the corresponding instruction manual. Biotinylated and fragmented cRNA targets (15 μg) were hybridized to Affymetrix Mouse Genome 430 2.0 arrays using the GeneChip Fluidics Station 450 according to the manufacturer's standard protocol. Arrays were scanned with a GeneChip Scanner 3000 7G, and the image data on each individual microarray chip were scaled to 150 target intensity, using the GeneChip Command Console Software (AGCC software v.1.1) (Affymetrix). Microarray data were analyzed in R (2.11.1) (R Core Team 2015) (http://www.R-project.org/) using the affy (1.26.1) (Gautier et al. 2004) and limma 3.4.3 (Smyth 2005) packages. Normalization was done using rma. Annotation information was taken from Bioconductor annotation package mouse4302.db (2.4.1). *k*-means clustering was done in *R* (2.13.2) with *k* = 9.

### Agilent tiling microarrays

Total RNA (1 µg) was amplified according to Ambion's Message Amp II aRNA Amplification Kit (AM1751). Agilent's One Color RNA Spike-In Kit was used as a positive control RNA. Amplified mRNA (aRNA) (2 µg) was labeled with cy3 dye and hybridized to custom Agilent 2x105K *Hox* tiling arrays (details in Supplemental Data) according to the manufacturer's instruction. Agilent tiling arrays were hybridized in a single-color configuration. Agilent “gMeanSignal” was used as the measurement for each spot. Data were analyzed using the limma package (Smyth 2005) and normalized between arrays using scale normalization. Replicates were averaged and bedGraph files were created and visualized using IGV (Thorvaldsdottir et al. 2013) and the UCSC Genome Browser (Kent et al. 2002).

### RNA-seq

Libraries were prepared using the Small RNA Sample Prep Kit (Illumina, FC-102-1010) with 10× v1.5 sRNA 3′ Adaptor (Illumina, 15000263) and mRNA-seq Library Prep Kit (Illumina, RS-100-0801) according to the manufacturer's protocol (15018460 Rev A Oct 10) (details in Supplemental Data) and sequenced.

### ChIP-on-chip

ChIP was done according to the Upstate protocol (Smith et al. 2010). Input DNA and IP DNA (10 ng) were amplified and labeled according to the Agilent Genomic DNA Labeling Kit PLUS (G4481-90010). Custom Agilent 2×105K *Hox* tiling arrays were hybridized with a mixture of 4 µg Cy3 labeled DNA and 4 µg Cy5 labeled DNA probes. Hybridizations were performed for 24 h at 65°C under standard conditions. Microarray images were acquired with an Agilent High-Resolution DNA Microarray Scanner (G2505C). For image analysis, Agilent Feature Extraction software (Version 10.5.1.1) was used. Agilent tiling arrays were hybridized in a two-color configuration. Data were analyzed using the limma (3.4.3) package and normalized within arrays using loess normalization.

### Quantitative PCR of *Hox* genes using TLDA cards

Quantative PCR was done using TLDA cards as described in Supplemental Methods. Analysis of the fluorescence curves was done using ABI's SDS2.3 software. All curves that showed errors as determined by the SDS2.3 software or that were above 35 Ct were thrown out. The remaining Ct values were exported and analyzed using DataAssist v2.0. *Gapdh* and *Tbp* were used as endogenous controls.

### Quantitative PCR of noncoding transcripts using SYBR green assays

For ES cell and mouse embryo RNA, 5 µg total RNA was used as a template in 20 µL total volume reaction of the SuperScript II (Invitrogen) reverse transcription kit using oligo dT primers. The 20-µL reaction was diluted 50 times, and 2 µL were used for qPCR and cycled on an ABI 7900HT according to ABI's standard protocol. Analysis of the fluorescence curves was done using ABI's SDS2.4 software. All curves that showed errors as determined by the SDS2.4 software or that were above 35 Ct were thrown out. The remaining Ct values were exported and analyzed using the Biogazelle qBase plus version 2.4 software to generate normalized relative quantities. *Gapdh* and *ATP5b* were used as endogenous controls. Each primer pair was standardized for a linear range of amplification through standard curve analysis. Analyses of the fluorescence curves was done using ABI's SDS2.4 software. All curves that showed errors as determined by the SDS2.4 software or that were above 35 Ct were thrown out. Analyses were done as discussed in the previous section.

### RA treatment of mouse embryos

Wild-type pregnant female CD-1 mice (10.0 dpc) and mice carrying the *DE-RARE* mutant alleles (Δ*DE*) (11.5 dpc) were orally injected (gavage) with all-*trans* retinoic acid dissolved in 160 µL of mineral oil to deliver a dose of 20 µg RA/g body weight. After 8 h, embryos were harvested and RNA was isolated. Total RNA (5 µg) was used as template in 50 µL total volume reaction of SuperScript II (Invitrogen) reverse transcription kit using oligo dT primers. Total RNA was isolated from whole embryos or pools of 3–5 dissected neural tubes and analyzed on an ABI 7900HT according to the standard protocol.

### Stellaris and HCR fluorescence in situ hybridization

Stellaris and HCR fluorescence in situ hybridization was done according to the manufacturer's protocol with minor modifications (details in Supplemental Methods). ES colonies were immobilized on 35-mm imaging grade plastic ibiTreat dishes (Ibidi Biosciences). Images were acquired with a Perkin-Elmer Ultraview spinning disc microscope with a CSU-X1 Yokogawa disc, equipped with a C9100 Hamamatsu Photonics EM-CCD. A 100× 1.4 NA Plan-apochromatic objective was used. The 405-nm, 561-nm, and 640-nm laser lines were used to excite DAPI, Quasar 570, and Quasar 670, respectively. Quasar 670 emission was collected through a 455–505 nm, 660–750 nm dual band pass filter, whereas Quasar 570 and DAPI were both collected with a 415–475 nm, 580–650 nm dual band pass filter.

## Data access

The raw and processed data from this study have been submitted to the NCBI Gene Expression Omnibus (GEO; http://www.ncbi.nlm.nih.gov/geo/) under series accession number GSE61590. All novel noncoding transcripts and isoforms described in this study have been assigned MGI-approved marker names with GenBank accession numbers as listed in Supplemental Table S7. All original source data for unprocessed microscope images and raw qPCR data have been deposited in the Stowers Institute Original Data Repository and are available online at http://odr.stowers.org/websimr/.

## Supplementary Material

Supplemental Material
