# Supplementary material for: Analysis of dynamic changes in retinoid-induced transcription and epigenetic profiles of murine Hox clusters in ES cells
Source: Genome Res. 2015 Aug;25(8):1229–43. doi: 10.1101/gr.184978.114 (PMC4510006; doi:10.1101/gr.184978.114)
Supplement: Supplemental Material [file supp_25_8_1229__index.html]

Analysis of dynamic changes in retinoid-induced transcription and epigenetic profiles of murine Hox clusters in ES cells — Analysis of dynamic changes in retinoid-induced transcription and epigenetic profiles of murine Hox clusters in ES cells — Supplemental Material 

# Analysis of dynamic changes in retinoid-induced transcription and epigenetic profiles of murine *Hox* clusters in ES cells

## Supplemental Material

**Files in this Data Supplement:**

- Supp Fig1.jpg
- Supp Fig2.ai
- Supp Fig3.jpg
- Supp Fig4.jpg
- Supp Fig5.jpg
- Supp Fig6.jpg
- Supp Fig7.jpg
- Supp Fig8.jpg
- Supp Fig9.jpg
- Supp Fig10.jpg
- Supplemental Information.docx
- Table S1.txt
- Table S2.txt
- Table S3.txt
- Table S4.txt
- Table S5.txt
- Table S6.docx
- Table S7.txt
