# Supplementary figures and images for: Analysis of dynamic changes in retinoid-induced transcription and epigenetic profiles of murine Hox clusters in ES cells
Source: Genome Res. 2015 Aug;25(8):1229–43. doi: 10.1101/gr.184978.114 (PMC4510006; doi:10.1101/gr.184978.114)

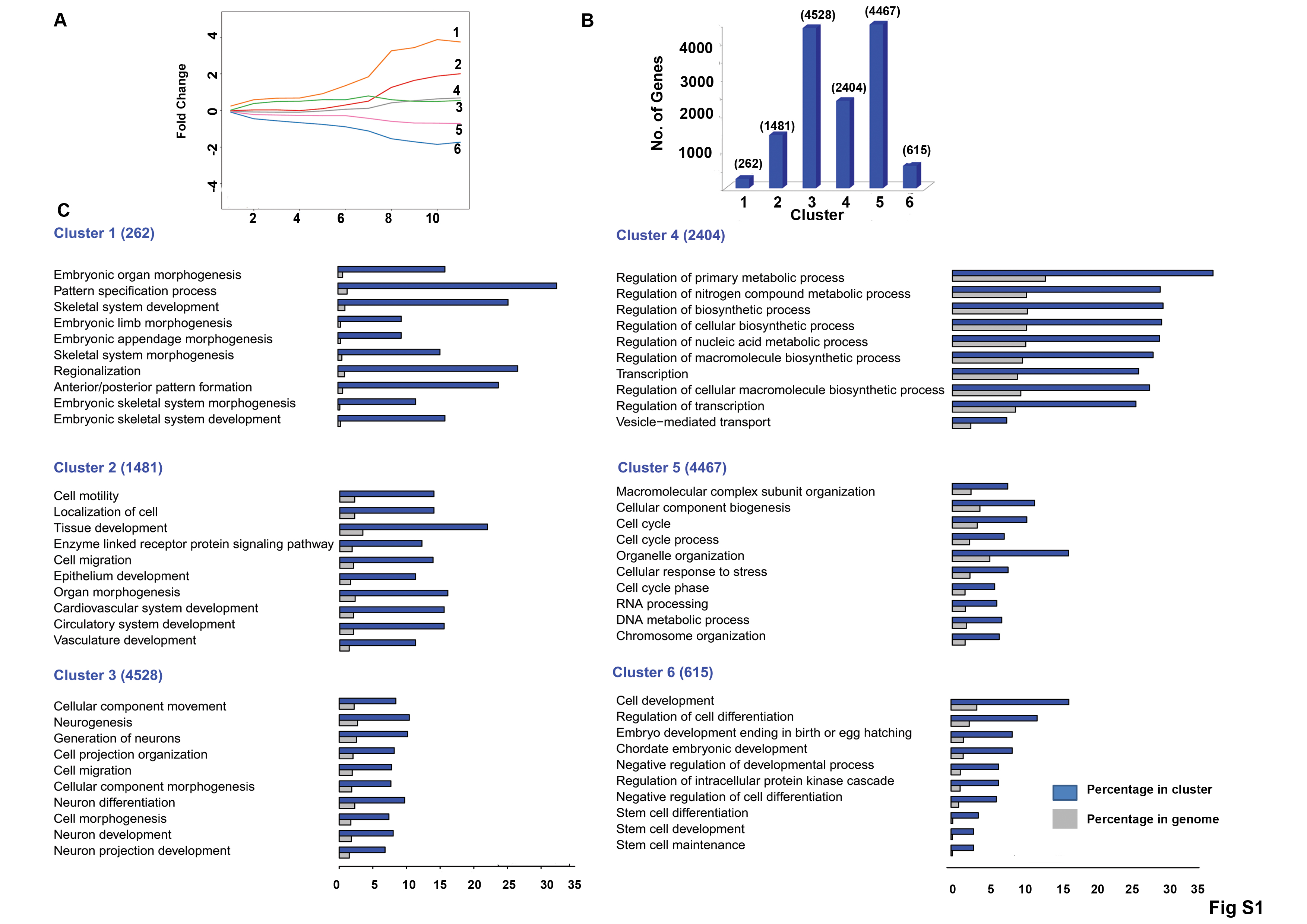

Supplement: Supplemental Material [file supp_gr.184978.114_Supp_Fig1.jpg]

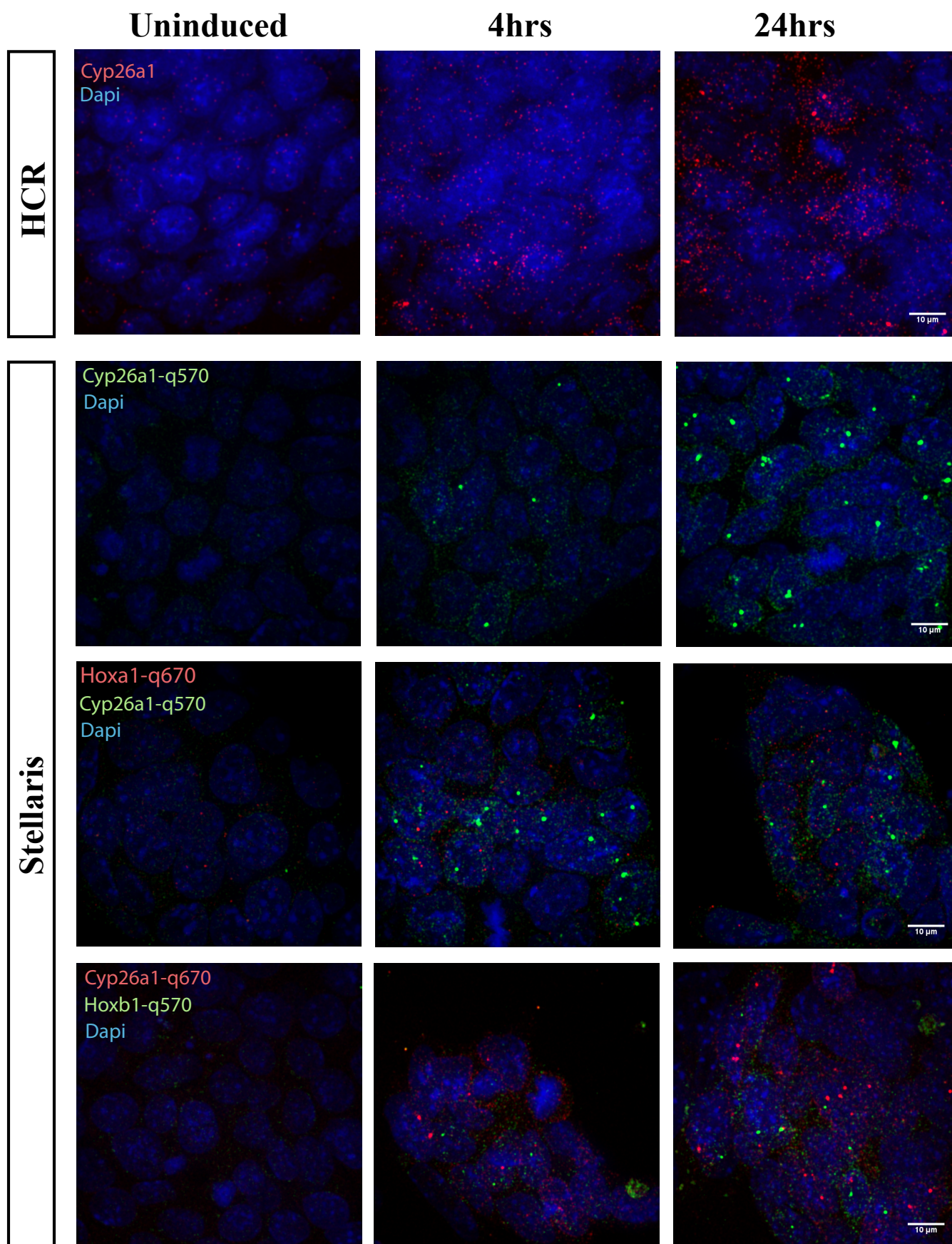

Fig S2

Supplement: Supplemental Material [file supp_gr.184978.114_Supp_Fig2.ps]

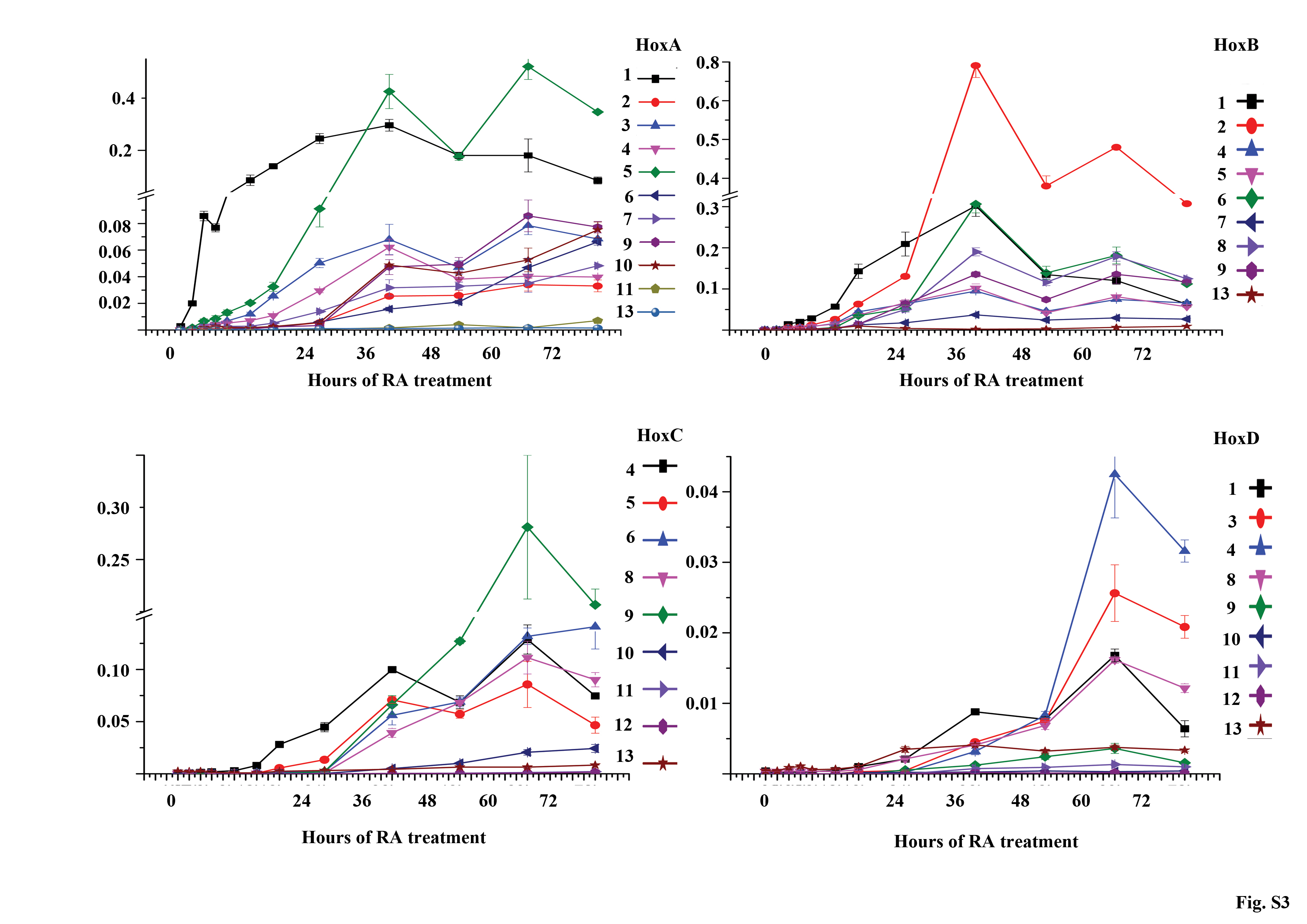

Supplement: Supplemental Material [file supp_gr.184978.114_Supp_Fig3.jpg]

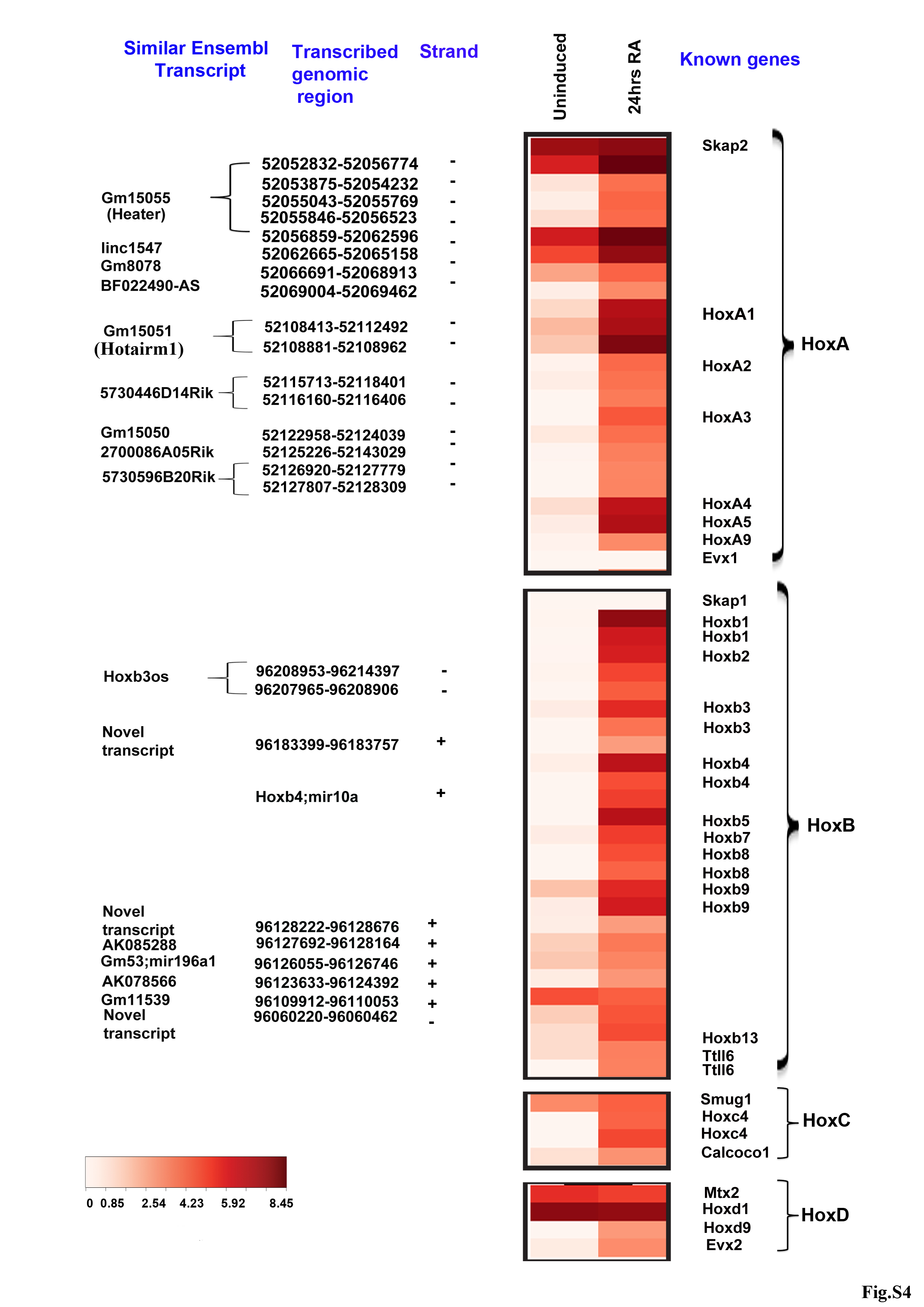

Supplement: Supplemental Material [file supp_gr.184978.114_Supp_Fig4.jpg]

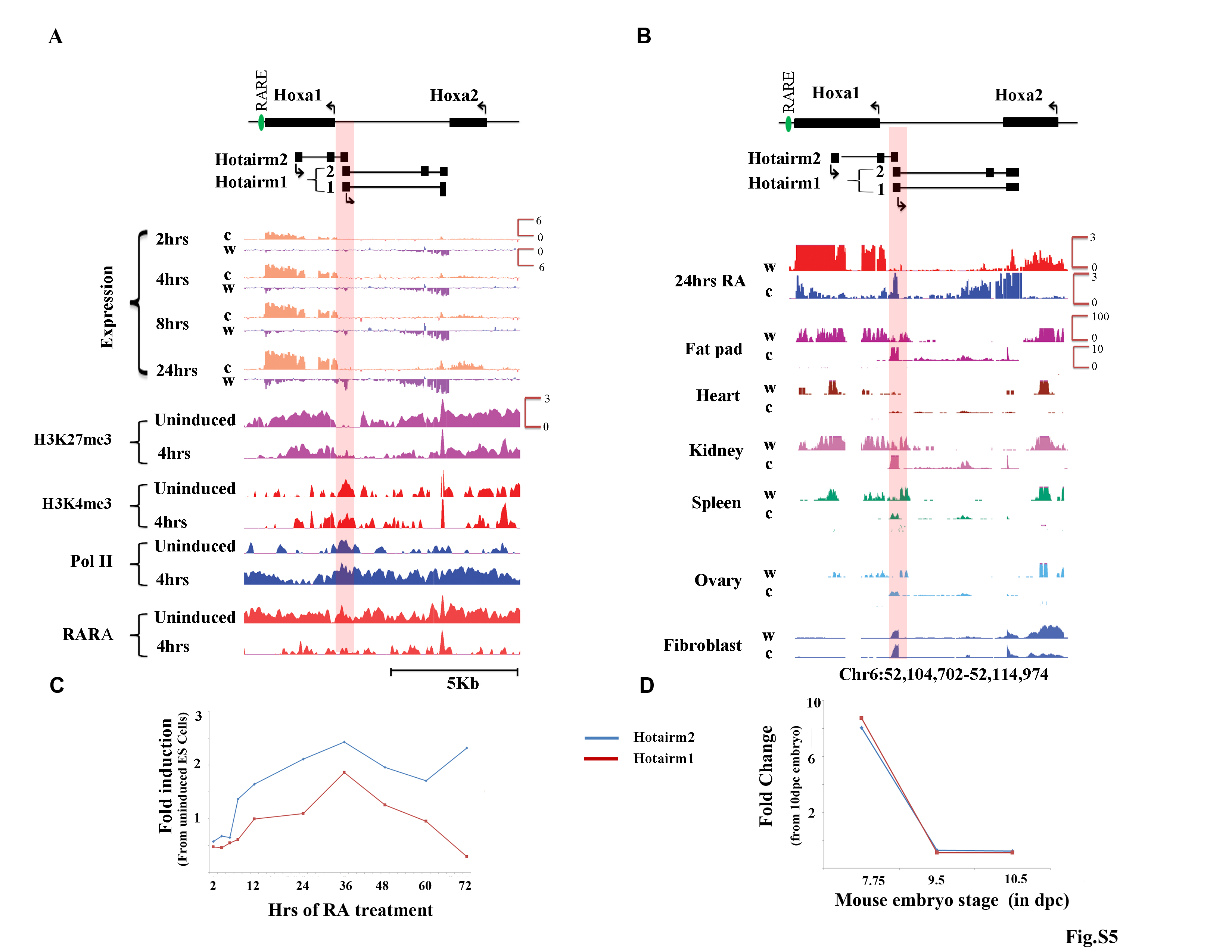

Supplement: Supplemental Material [file supp_gr.184978.114_Supp_Fig5.jpg]

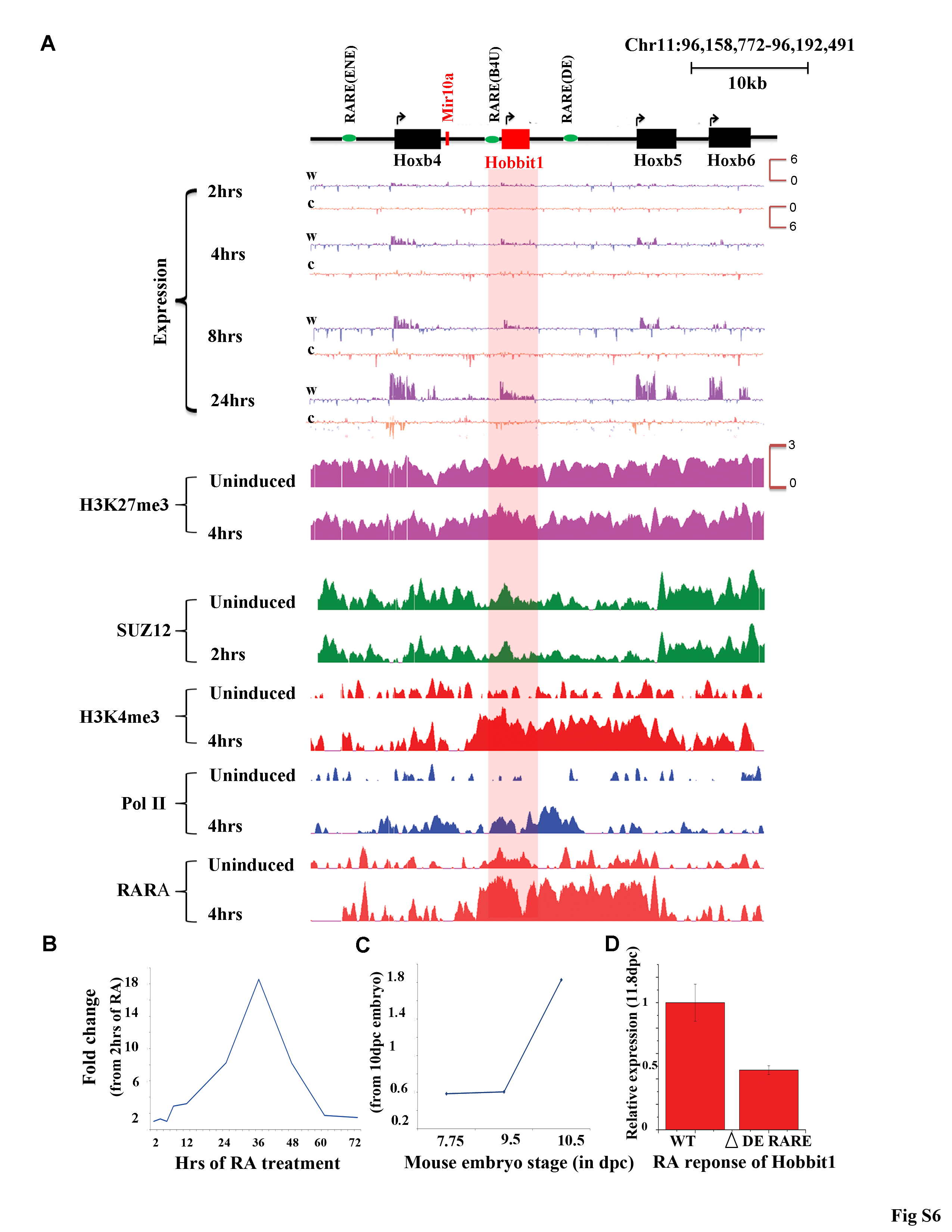

Supplement: Supplemental Material [file supp_gr.184978.114_Supp_Fig6.jpg]

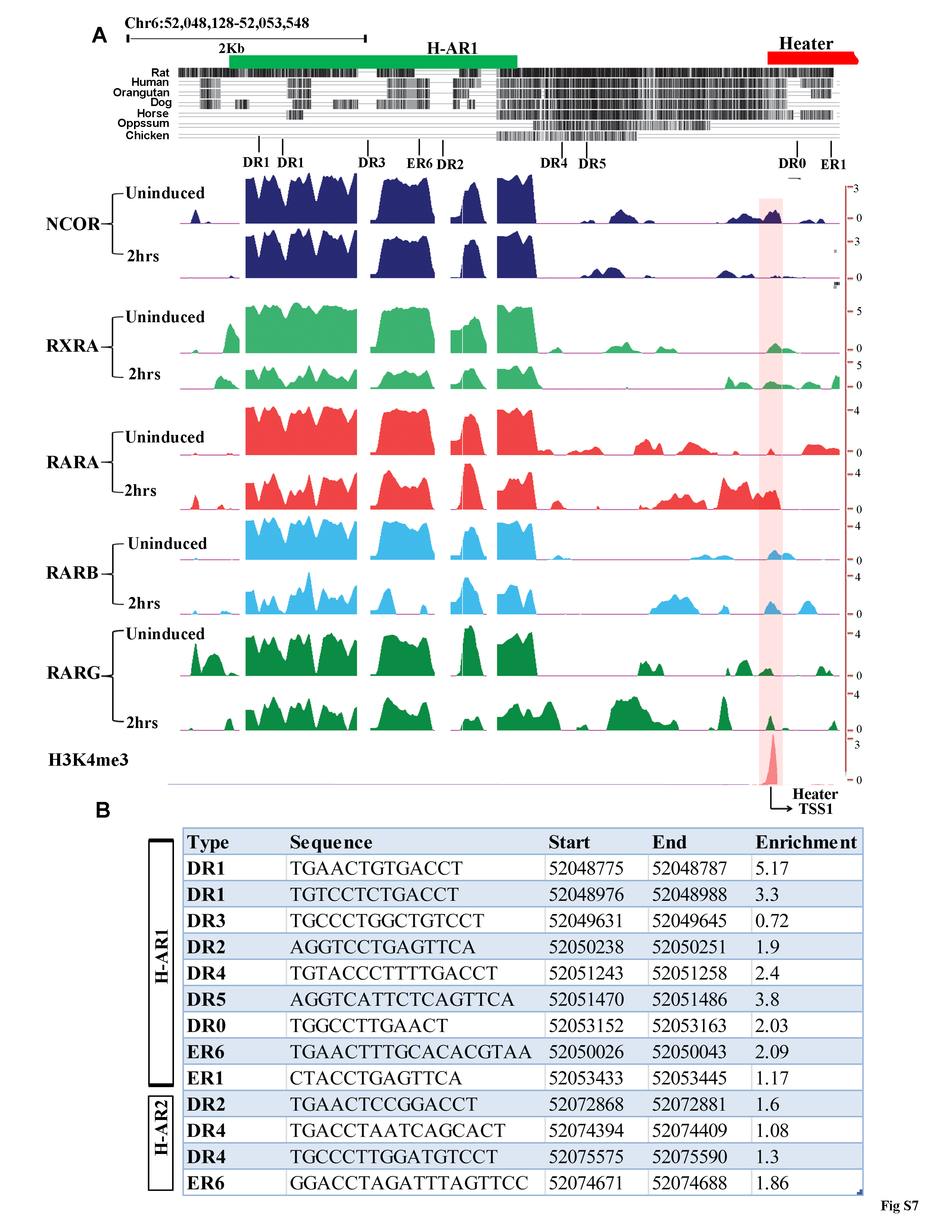

Supplement: Supplemental Material [file supp_gr.184978.114_Supp_Fig7.jpg]

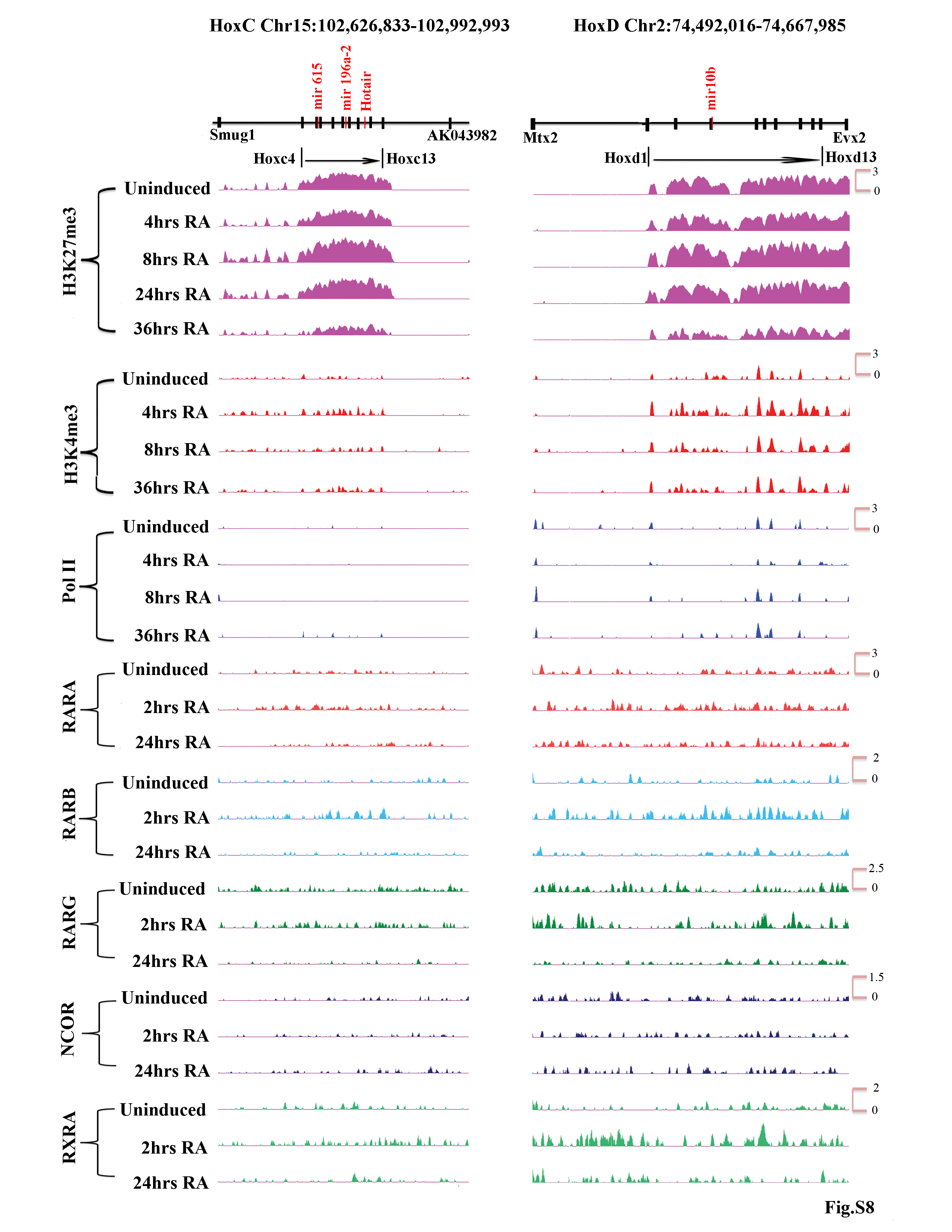

Supplement: Supplemental Material [file supp_gr.184978.114_Supp_Fig8.jpg]

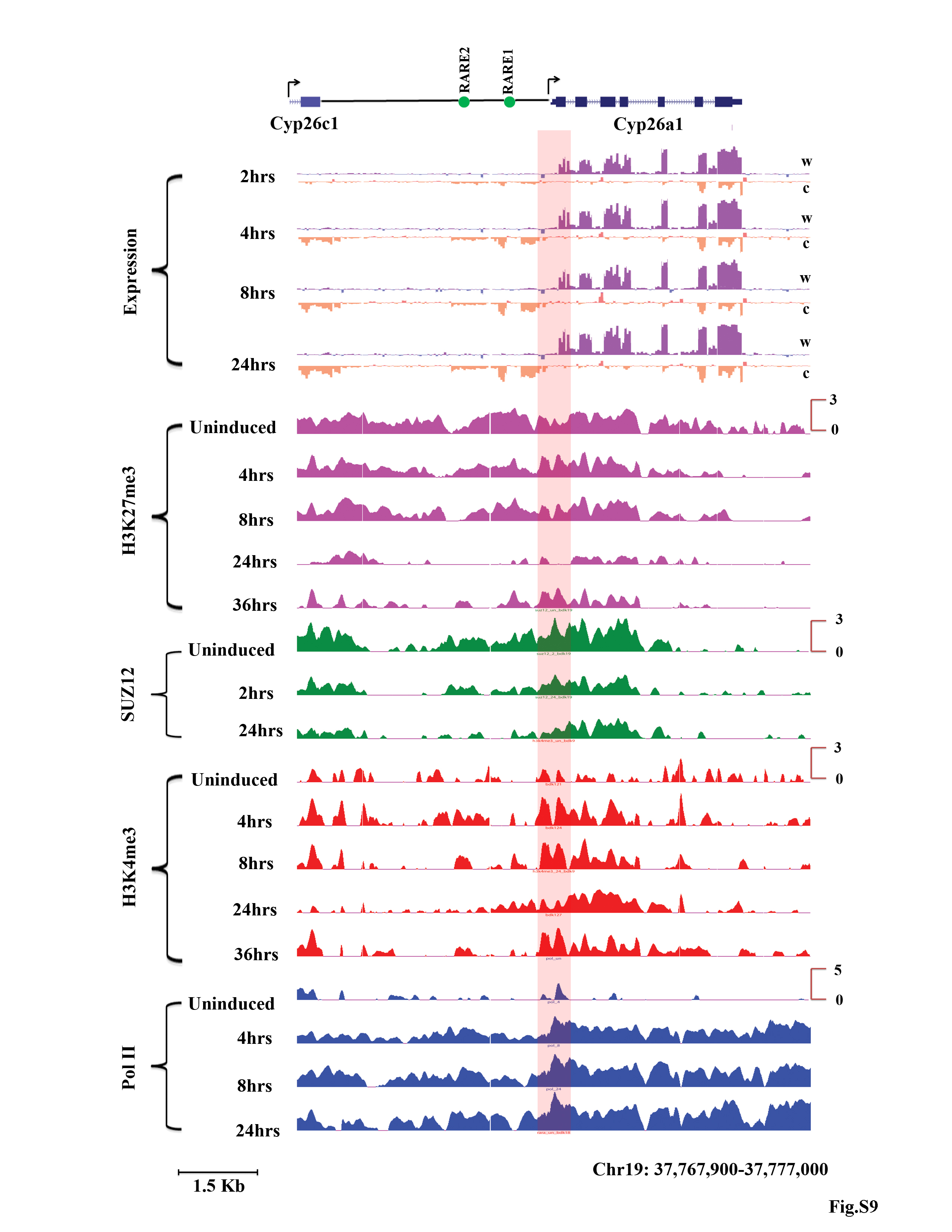

Supplement: Supplemental Material [file supp_gr.184978.114_Supp_Fig9.jpg]

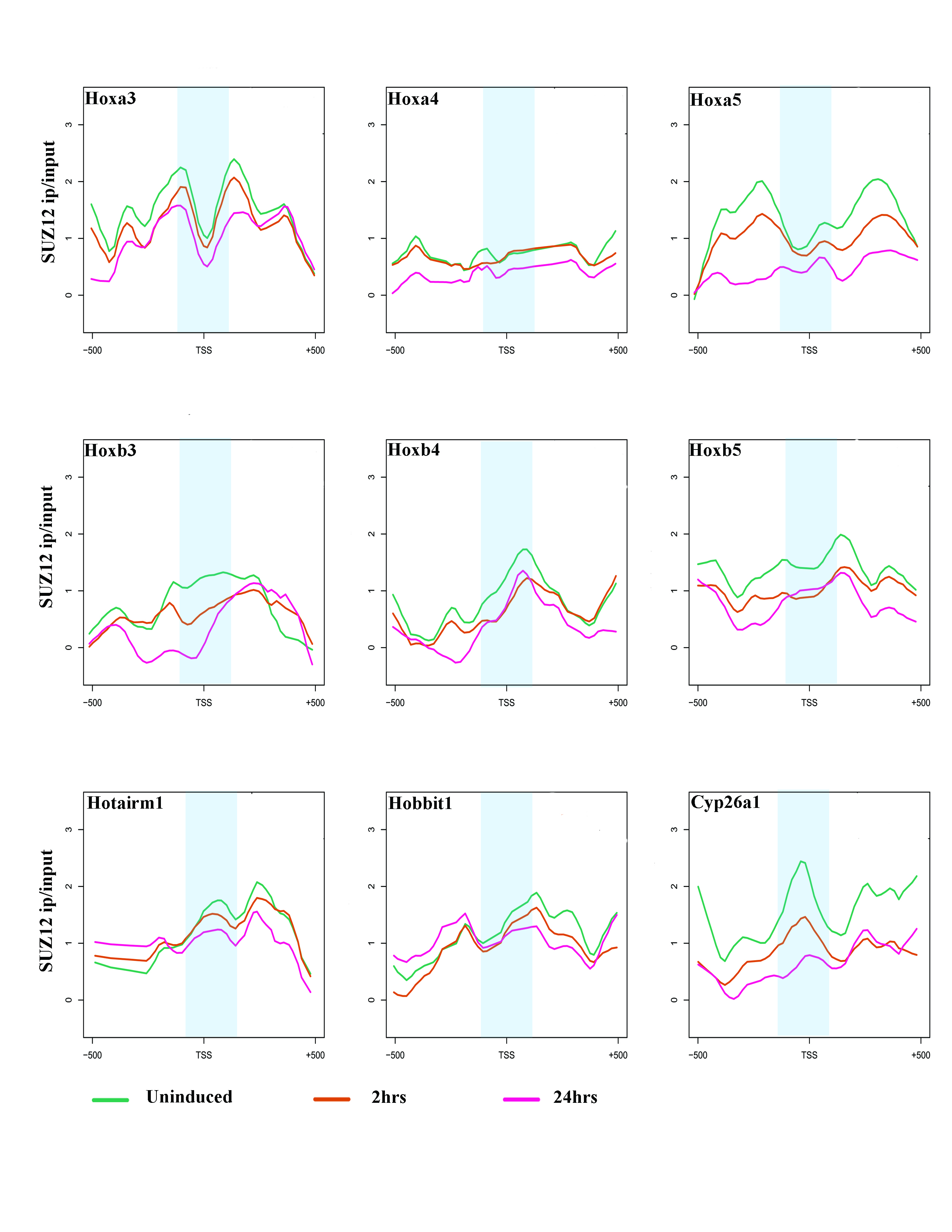

Supplement: Supplemental Material [file supp_gr.184978.114_Supp_Fig10.jpg]
