# Supplementary material for: Analysis of dynamic changes in retinoid-induced transcription and epigenetic profiles of murine Hox clusters in ES cells
Source: Genome Res. 2015 Aug;25(8):1229–43. doi: 10.1101/gr.184978.114 (PMC4510006; doi:10.1101/gr.184978.114)
Supplement: Supplemental Material [file supp_gr.184978.114_Supplemental_Information.docx]

**Supplementary Information for article:**

***Analysis of dynamic changes in retinoid induced transcription and epigenetic profiles of murine Hox clusters in ES cells***

Bony De Kumar, Mark E. Parrish, Brian D Slaughter, Jay R Unruh, Madelaine Gogol, Christopher Seidel, Ariel Paulson, Hua Li, Karin Gaudenz, Allison Peak, William McDowell, Brian Fleharty, Youngwook Ahn, Chengqi Lin, Edwin Smith, Ali Shilatifard and Robb Krumlauf

**Supplementary data includes: 10 supplementary figures & 7 Supplementary tables**

**Figure S1**. Gene ontology (GO) term analysis of differentially expressed genes. (A) *k*-mean clusters and their induction profile. (B) Number of genes in each of the six clusters. (C) Enriched GO terms for each cluster. Percentage of terms in each cluster (blue bars) and the whole genome (gray bars) are indicated. The X-axis indicates the percentage of terms in each cluster or the genome.

**Figure S2**. Stellaris and HCR FISH images of gene expression. Single and dual color images are shown as a max projection of 10 binned slices– spanning 6 microns. For each row of the FISH channels the contrast is set identically to allow for direct comparison of intensity between uninduced and induced colonies. The 1-2 large bright dots within nuclei represent nascent transcripts while the small dots dispersed over the nucleus and cytoplasm represents mature transcripts. Contrast is boosted for FISH channels to allow visualization of both the clustered nascent nuclear transcripts and dispersed single mature transcripts, giving the false appearance of saturation of nascent transcripts. A handful of dots are apparent in uninduced cells. This may represent expression in a small proportion of differentiated cells in the population, a low basal level of transcription in selected uninduced cells or a very low level of non-specific background. DAPI staining is variable and contrast adjustments are made for this channel.

**Figure S3.** Quantitation of changes in *Hox* gene expression upon RA induced differentiation using Applied Biosystems TLDA (Taqman Low Density Array) cards for qPCR. Non-scaled Ct values were normalized against Ct values of *Gapdh* and *Tbp*. All data points are average of three biological and two technical replicates. The Y-axis represents absolute change in expression, while the X-axis represents length of RA treatment in hours. Due to differences in relative levels of induction between the four *Hox* clusters, the Y-axes are displayed using different scales to illustrate temporal kinetics.

**Figure S4.** Quantitative expression analysis of genes and non-coding transcripts in and around the four *Hox* clusters using RNA-seq. Expression of transcripts from uninduced ES Cells and those treated for 24 h with RA are compared. Average FPKM values from two biological replicates are used for generation of a heat map. Known genes within the clusters are indicated at the right, while on the left, non-coding transcripts are noted along with their coordinates. Fewer transcripts were observed from *HoxC* and *HoxD* clusters compared to *HoxA* and *HoxB*.

**Figure S5.** Characterization of the non-coding *Hotairm1* and *Hotairm2* transcripts. (A) Expression and epigenetics changes in *Hoxa1-a2* intergenic region. Rapid induction of *Hoxa1*, *Hotairm1* and *Hotairm2* between 2-24 h of RA treatment is indicated by tiling array profiles. Two isoforms of *Hotairm1* are shown schematically with the direction of transcription indicated by arrows. ChIP on chip analysis in this region indicates an increase of H3K4me3and Pol II and rapid loss of H3K27me3 and RARA by 4 h of RA treatment**.** (B) Evidence of *Hotairm1* and *Hotairm2* in adult tissues based on ENCODE data (Rosenbloom et al. 2013)**.** In A and B: the green oval 3’ of *Hoxa1* refers to an RARE required for its regulation in neural tissue and w and c represent Watson and Crick strands respectively. (C) qPCR quantification of *Hotairm1* and *Hotairm2* transcripts in differentiating ES cells. X-axis represents length of RA treatment in hours. (D) qPCR quantification of *Hotairm1* and *Hotairm2* transcripts in developing mouse embryos. X-axis represents three different stages of mouse embryonic development. Y-Axis in C and D shows fold induction compared to uninduced ES cells and 10 dpc embryo respectively. Both *Hotairm1* and *Hotairm2* transcripts show similar induction kinetics.

**Figure S6.** Characterization of the non-coding *Hobbit1* transcript. (A) Expression and epigenetic changes in *Hoxb4-b5* intergenic region. Rapid induction of *Hoxb4, Hoxb5* and *Hobbit1* between 2-24 h of RA treatment is indicated by tiling array profiles. ChIP on chip analysis of this intergenic region at 4 h of RA treatment indicates a rapid gain of H3K4me3, Pol II and RARA occupancy and little change in H3K27me3 repressive mark. However, there is a reduction in occupancy of the SUZ12 PRC2 associated repressive mark over the *Hobbit1* region at 2 h of RA treatment. The schematic drawing at the top indicates the relative positions of *Hox* genes and *Hobbit1* and the arrows represent direction of transcripts. The green ovals refer to three previously characterized RAREs (*ENE, DE* and *B4U*) involved in *HoxB* regulation; and w and c represent Watson and Crick strands respectively. (B) qPCR quantification of the *Hobbit1* transcript in differentiating ES cells. X-axis represents length of RA treatment in hours. (C) qPCR quantification of the *Hobbit1* transcript in developing mouse embryos. X-axis represents three different stages of mouse embryonic development. Y-Axis in B and C shows fold induction compared to uninduced ES Cells and 10 dpc embryos respectively. (D) RA response of *Hobbit1* in wild type and *DE- RARE* mutant embryos. The RA response is calculated based on qPCR as fold change in relative expression levels after RA gavage by comparing dissected hindbrains of wild type and *DE-RARE* homozygous null mouse embryo littermates.

**Figure S7** Characterization of *H-AR1* displaying a high level of occupancy of retinoid receptors.  **(**A) Occupancy of retinoic acid receptors (RXRA, RARA, RARB, and RARG) and the NCOR co-repressor in the *H-AR1* domain upstream of *Heater*. ChIP on chip analysis shows a broad 2.5 kb region bound by RAR/RXRs and NCOR in uninduced ES cells and cells treated for 2 h with RA. At the top is an alignment showing conservation of this region among selected vertebrates and the relative positions of predicted consensus Direct Repeat motifs recognized by retinoid receptors. (B) List of predicted retinoid receptor Direct Repeat motifs found in *H-AR1* and *H-AR2* using the NHR Scan program, along with precise sequences and genomic coordinates.

**Figure S8**. ChIP on chip analysis of changes in the epigenetic state and retinoid receptor occupancy of *HoxC* and *HoxD* clusters during RA induced differentiation. H3K27me3 is used as a repressive mark, H3K4me3, a mark for an active chromatin state and Pol II, as a mark of active transcription. Tracks are configured by using windowing function as mean and smoothing windows as 0 pixels in UCSC Genome Browser. All time points for a given antibody are normalized with same Y-axis and the specific range of each Y-axis is shown for respective uninduced samples. Schematic at the top indicates the relative positions of *Hox* genes, microRNAs and non-coding transcripts. There are relative few epigenetic changes observed over these clusters during the time course as genes in *HoxC* and *HoxD* have a delayed respond to RA compared with *HoxA* and *HoxB*. However, H3K27me3 is greatly reduced by 36 h of RA treatment.

**Figure S9.** Characterization of expression and epigenetic changes of *Cyp26a1*. Rapid induction of *Cyp26a1* between 2-24 h of RA treatment is indicated by tiling array profiles. ChIP on chip analysis of this region at 4 h of RA treatment indicates a rapid gain of H3K4me3 and Pol II occupancy but little change in H3K27me3 repressive mark. There is a reduction in occupancy of the SUZ12 PRC2 associated repressive mark by 2 h. The schematic drawing at the top indicates the relative positions of *Cyp26a1* and *Cyp26c1* genes and the arrows represent direction of transcripts. W and C represent Watson and Crick strands respectively.

**Figure S10.** Kinetics of reduction of SUZ12 occupancy over TSS of *HoxA* and *HoxB* genes in paralogy groups 3-5, *Hobbit1*, *Hotairm1* and *Cyp26a1* during RA induced differentiation of ES cells. The differences in the kinetics of loss of SUZ12 between genes correlates with their respective time of activation, illustrated by the reduction of SUZ12 for *Hoxb4* and *Hoxb5* at 2 h of RA treatment. A 500 bp region around the TSS is shown and a 50 bp region around TSS is marked by a light blue band. Y-axis shows relative levels of occupancy of SUZ12.

**Table S1**. Affymetrix gene expression changes during RA induced differentiation of ES cells. All values shown are compared with uninduced ES cells. Both probe IDs and gene names are given for clarity.

**Table S2.** Quantification of coding and non-coding transcripts from *Hox* clusters using RNA–seq of uninduced and 24 h RA induced ES cells. Transcript structures are generated by Cufflink and Cuffcompare. Loci detected in only one technical replicate have been removed. FPKM values from both biological replicates are shown in this table. Columns listed include: Gene, Chr, Start, End, Strand, Uxons, RPKM values (24 h RA treated and uninduced ES cells) and Gene models.

**Table S3.** Quantification of *Heater (Halr1 and Halr1os1)*, *Hotairm1, Hotairm2* , *Hobbit1* and *Hox* genes in differentiating ES cells and developing embryos. Cufflinks unguided runs were carried out to generate *de-novo* transcript and gene models. The Cufflinks models for regions overlapping the *Hobbit1* and *Heater* were added to Ensembl78 GTF. All samples were re-quantitated using Cufflinks and the modified Ensembl78 GTF. Assays were done with RNA from developing embryos (9.5 dpc and 10.5 dpc), uninduced ES cells and following 4 h, 6 h, 12 h, 24 h and 36 h of RA treatment.

**Table S4.** Quantification of non-coding Transcripts using q-PCR. Mean fold change and value higher and lower than 95% confidence is also shown in the table. Mean calculated in this table is geometric mean.

**Table S5**. Genes and coordinates of probes for non-*Hox* genes in tiling arrays.

**Table S6.** Details of primers for qPCR quantification of non-coding transcripts. Sequence of forward and reverse primers and genomic coordinates are shown. Amplicon size is indicated in parenthesis.

**Table S7**. Genetic marker names and GenBank accession IDs for transcripts and their isoforms.

**Extended Methods**

**Induction of KH2 cells with retinoic acid**

KH2 cells at passage 12 were cultured on gamma irradiated feeder cells with DMEM containing 15% fetal bovine serum, NEAA, β-mercaptoethanol and LIF. ES Cells were differentiated with differentiation media (DMEM + 10% Serum + NEAA+ 0.03 µM RA) RA supplemented 33nm for requisite length of time. Feeders were separated by plating freshly trypsinized cells on a gelatinized plate for 30 min. After half an hour media was aspirated and centrifuged for 5 min at 1000 rpm. Pellets were dislodged by gentle tapping and 2 ml TRIzol was added. These tubes were stored at -80 until RNA isolation. RNA isolation was performed using TRIzol and later purified by the RNeasy Kit (Qiagen). RNA was tested for integrity and concentration using the RNA 6000 Nano Assay and RNA LabChips on the Agilent Bioanalyzer 2100 (Agilent Technologies, Inc., Palo Alto, CA)

**RNA preparation for Affymetrix microarray analysis**

200 ng total RNA was labeled and cRNA targets were generated from total RNA samples using the MessageAmp III RNA Amplification Kit (Applied Biosystems/Ambion, Austin, TX.) according to the corresponding instruction manual. Biotinylated and fragmented cRNA targets (15 μg) were hybridized to Affymetrix Mouse 430 v2 arrays using the GeneChip Fluidics Station 450 according to the manufacturer’s standard protocol. Arrays were scanned with a GeneChip Scanner 3000 7G and the image data on each individual microarray chip was scaled to 150 target intensity, using the GeneChip Command Console Software (AGCC software v.1.1) (Affymetrix, Santa Clara, CA). Microarray data was analyzed in R (2.11.1) (URL http://www.R-project.org/) using the Affy (1.26.1) (Gautier et al. 2004) and limma 3.4.3 (Smyth 2005) packages. Normalization was done using rma. Annotation information was taken from Bioconductor annotation package mouse 4302.db (2.4.1). k-means clustering was done in R (2.13.2) with k = 9.

**Design and analysis of Agilent tilling microarrays**

Custom made tilling arrays were generated with probes designed as 60mers overlapping by 20 bases, on both strands. Probes matching more than one place in the genome with 100% identity (aligned with Blat) were removed; so on average there is one probe every 22 bases. Design number- 027887 "rek1" (2x105k Agilent array). Regions covered are (mouse genome version mm9) : *HoxA* (chr6:51960548-52269383), *HoxB* (chr11:95994533-96346113), *HoxC* (chr15:102626926-102985721) and *HoxD* (chr2:74351893-74665608) which is equivalent to : for *HoxA* cluster -1000 bases into *Skap2* to and past *Evx1* for *HoxB* cluster -1000 bases past *Ttll6* into *Skap1*, for *HoxC* cluster- 1000 bases past *AK043982* to 1000 bases into *Smug1* and for *HoxD* cluster- 1000 bases past *Lnp* to 1000 bases into *Mtx2*. 94 non-*Hox* genes with their flanking regions were also included in this array (Table S5).

**RNA preparation, hybridization and analysis of Agilent tiling microarrays**

RNA isolation was performed using TRIzol and later purified by RNeasy Kit (Qiagen). Total RNA in the amount of 1µg was amplified according to Ambion’s Message Amp II aRNA Amplification Kit, part number AM1751. Positive control RNA from Agilent’s One Color RNA Spike-In Kit, part number 5188-5282, was used to monitor sample amplification and labeling as well as array hybridization. Amplified mRNA, referred to as aRNA, was quantified on a NanoDrop ND-1000 and a mass of 2 µg was labeled with cy3 dye using Kreatech’s ULS Fluorescent Labeling Kit, part number EA-023. Labeled aRNA was quantified on the NanoDrop ND-1000 and a 1.5 µg mass of cy3 labeled aRNA was hybridized to custom Agilent 2x105K Hox tiling arrays. Hybridizations were performed at 65^o^C for 17 h under standard conditions (1X Agilent blocking agent, and 1X Agilent hybridization buffer) and slides were washed successively with Agilent GE wash buffer 1, at room temperature and with Agilent GE wash buffer 2, at 31^o^C, prior to scanning. Microarray images were acquired with an Agilent High-Resolution DNA Microarray Scanner (G2505C). Hybridization, array washing, scanning and probe information extraction with Agilent’s Feature Extraction Software (Version 10.5.1.1) were all performed according to Agilent’s One-Color Microarray-Based Gene Expression Analysis Protocol, Version 6.0, December 2009 (Low Input Quick Amp Labeling). In analysis of expression, Agilent tiling arrays were hybridized in a single-color configuration and data was read into R. Agilent "gMeanSignal" was used as the measurement (mean green signal for each spot). Data was analyzed using the limma package (Smyth 2005). Data was normalized between arrays using scale normalization. Replicates were averaged and bedgraph files were created and visualized using IGV (Thorvaldsdottir et al. 2013) and UCSC Genome Browser (Kent et al. 2002).

**RNA-seq library preparation, sequencing and analysis**

RNA isolation was performed using TRIzol and later purified by RNeasy Kit (Qiagen). Quality of RNA was analyzed by Agilent 2100 Bioanalyzer. RNA with more than 9 RIN (RNA integrity number) were used in library preparation. Libraries were prepared using the Small RNA Sample Prep Kit (Illumina, FC-102-1010) with 10x v1.5 sRNA 3’ Adaptor (Illumina, 15000263) and mRNA-Seq Library Prep Kit (Illumina, RS-100-0801) according to manufacturer’s mRNA-Seq Sample Prep protocol (15018460 Rev A Oct 10). Briefly, 1 µg Total RNA was enriched for poly(A)+ RNA by oligo(dT) selection.  The Poly(A)+ RNA was then fragmented, and the ends repaired using phosphatase and PNK treatments. Illumina’s v1.5 sRNA 3’ adaptor was ligated to the blunted RNA segment, followed by the ligation of the kit’s standard SRA 5’ adaptor. The libraries were reverse transcribed and enriched by 15 rounds of PCR. The purified libraries were quantified with the High Sensitivity DNA assay on an Agilent 2100 Bioanalyzer and. Libraries were sequenced single read with 36 nt sequencing on a GAIIx and fastq files were returned .

For each sample, reads were aligned to mm9 using Tophat 1.4.1 and the Ensembl 65 GTF, allowing uniquely-aligning reads only (parameters: -g 1 -x 1 --segment-length 20 --segment-mismatches 2). Initial BAM files were split into separate BAM files for + and – strand alignments. Strand-wise BAM files were quantitated with Cufflinks 1.3.0 and no GTF (parameters: --max-mle-iterations 10000 --max-bundle-frags 100000000 -F 0.05 -a 0.05 --trim-3-dropoff-frac 0.01). For each time point and strand, *ab-initio* cufflinks transcripts were condensed into loci using Cuffcompare 1.3.0, default parameters, using the above GTF file as the reference. Any such *ab-initio* loci which were detectable in only one technical replicate were removed. Genomic coordinates for the four Hox regions were extended out to their proximal flanking genes, resulting in the following regions of interest: *HoxA* (chr6:51809165-52268372), *HoxB* (chr11:95995100-96620791), *HoxC* (chr15:102537210-102988383) and *HoxD* (chr2:74491049-74716488). All *ab-initio* loci from these regions were extracted and displayed as a heatmap. Loci were arranged in genomic order. For each time point, heatmap values are log2 of the average FPKM of the Cuffcompare transcripts assigned to that locus.

For identification of novel transcripts from ncRNAs described in this study, strand specific libraries from ribo-depleted RNA of uninduced ES cells, RA treated ES cells (4 h, 6 h, 12 h, 24 h and 36 h) and developing embryos (9.5 and 10.5 dpc) were sequenced as 50 bp single end read on HiSeq2500. Cufflinks unguided runs were done on all samples (no GTF used). This produced *de-novo* transcript models and gene models. Cufflinks models for regions overlapping the *Hobbit1* or *Heater* were added to Ensembl 78 GTF. One Ensembl model was removed, *Hoxb5os* (ENSMUSG00000085645), because its transcripts were not recovered from assembly, and also because other *Hobbit1*-overlapping transcripts existed which directly competed with it. All samples were re-quantitated using Cufflinks and the modified Ensembl 78 GTF.

**ChIP on chip**

ChIP was done according to the Upstate protocol with certain modification (Smith et al. 2010).

Antibodies used were: Abcam: H3K4me3 (Ab1012), H3K27me3 (Ab6002), RARA (Ab28767),

RARB (Ab53161), RARG (Ab97569), NCOR1/2 (Ab24552) and SUZ12 (Ab12073); Santa Cruz Biotechnology, Inc.: Pol II (Sc889) and RXRA (Sc-553X); and custom made antibodies for AFF4, CDK9 and ELL2 as described in (Lin et al. 2011). Cells were fixed by adding formaldehyde to media at a final concentration of 1% and by incubating at 37^o^C for 11 min. Crosslinking was stopped by 1mL 1.25 M glycine per 10 mL and by incubating at room temperature for 5 min. Cells were sonicated for 25 min in Bioruptor at high setting and 30 sec on-off cycle. Input DNA and IP DNA in the amount of 10 ng were amplified and labeled according to the Agilent Genomic DNA Labeling Kit PLUS (Agilent, 5188-5309, Agilent publication number G4481-90010). Custom Agilent 2x105K Hox tiling arrays were hybridized with a mixture of 4 µg Cy3 labeled DNA and 4 µg Cy5 labeled DNA probes. Hybridizations were performed at 65^0^C for 24 h under standard conditions (45 mg/mL Human Cot-1 DNA, 1X Agilent blocking agent, and 1X Agilent hybridization buffer) and slides were washed successively with Agilent ChIP wash buffer 1, at room temperature and then Agilent ChIP wash buffer 2, at 31C, prior to scanning. Microarray images were acquired with an Agilent High-Resolution DNA Microarray Scanner (G2505C). For image analysis Agilent Feature Extraction software (Version 10.5.1.1) was used. Agilent tiling arrays were hybridized in a two-color configuration and data was read into R (2.11.1). Data was analyzed using the limma (3.4.3) package. Data was normalized within arrays using loess normalization.

**Quantitative PCR of *Hox* genes using Applied Biosystems TLDA cards**

400ng aliquots of each RNA sample were used as the template in 20 µl total volume reactions of ABI’s High Capacity cDNA Reverse Transcription Kit. These 20 µl reactions were combined with 80 µl ultrapure water and 100 µl of ABI 2x Gene Expression Master Mix. For each of the resulting reactions, 95 µl was added to 2 lanes of a 48 assay Custom TLDA Card from ABI. The TLDA cards were spun down, sealed, and cycled on an ABI 7900HT according to ABI’s standard protocol. Analysis of the fluorescence curves was done using ABI’s SDS2.3 software. All curves that showed errors as determined by the SDS2.3 software or that were above 35 Ct were thrown out. The remaining Ct values were exported and analyzed using DataAssistv2.0. DataAssistv2.0 was used to determine the most stably expressed endogenous control genes. *Gapdh* and *Tbp* were used as endogenous controls.

**Characterization of Non-coding transcripts**

Based on signals from tilling array, evidence for transcripts were searched in various databases like RefSeq, Ensembl and genome wide sequencing studies. Previous genome-wide transcriptome identification and prediction studies identified several transcripts including ENSMUST00000125375, ENSMUST00000155758, ENSMUST00000121011, MTR048842.6.884.0, MTR048842.6.884.1, MTR048842.6.884.2 and *Halr1* (*Hox* adjacent long non-coding RNA) in *Heater* region while ENSMUST00000129546 , ENSMUST00000132559 and ENSMUST00000159006 were identified for *Hotairm1* and *Hotairm2*. Based on structure of these transcripts and tilling array data a series of forward and reverse primers were designed for primer walking. Parts of transcripts were amplified, cloned and sequenced. Based on sequencing data obtained from primer walking, transcript structure were built and using 5’ RACE ends of transcripts were identified.

**Quantitative PCR of Non-coding transcripts using syber green assays**

For ES cell and mouse embryo RNA 5 µg total RNA was used as template in 20 µl total volume reaction of Superscript II (Invitrogen) reverse transcription kit using oligo dT primers. 20 µl reaction was diluted 50 times and 2 µl were used for qPCR and cycled an ABI 7900HT according to ABI’s standard protocol. Analysis of the fluorescence curves was done using ABI’s SDS2.4 software. All curves that showed errors as determined by the SDS2.4 software or that were above 35 Ct were thrown out. The remaining Ct values were exported and analyzed using the Biogazelle qBase plus version 2.4 software to generate normalized relative quantities. *Gapdh* and *Atp5b* were used as endogenous controls. Each primer pair were standardized for a linear ranges of amplification through standard curve analysis (Table S6). Analysis of the fluorescence curves was done using ABI’s SDS2.4 software. All curves that showed errors as determined by the SDS2.4 software or that were above 35 Ct were thrown out. Analysis were done as discussed in previous section.

**RA treatment of mouse embryos by gavage and analysis**

In general, wild type pregnant female CD-1 mice carrying embryos at 10.0 dpc were orally injected (gavage) with all-trans retinoic acid dissolved in 160 µl of mineral oil to deliver a dose of 20 µg RA/g body weight. After 8 h, embryos were harvested and RNA isolated by the TRIzol method. 5 µg total RNA was used as template in 50 µl total volume reaction of Superscript II (Invitrogen) reverse transcription kit using oligo dT primers. For RA gavage in mice carrying the *DE-RARE* mutant alleles (*ΔDE*) (Ahn et al. 2014), RA gavage was given to pregnant females carrying 11.5 dpc embryos from a cross of *+/ DEΔ* F1 animals. After 8 h anterior neural tubes from the midbrain–hindbrain boundary to the level of the third dorsal root ganglion (C3) were dissected out from homozygous mutant embryos and wild type littermates. For gene expression analysis, total RNA was isolated from pools of 3 to 5 dissected neural tubes using TRIzol (Life Technologies). For qPCR 20 µl of RT reactions was diluted 10 times and 2 µl were used for qPCR and cycled an ABI 7900HT according to ABI’s standard protocol.

**Stellaris and HCR fluorescent *in situ* hybridization**

For the Stellaris protocol probe sequences for single molecule RNA FISH for *Hoxa1, Hoxb1*, and *Cyp26a1* mRNA sequences were generated with the Biosearch Technologies Stellaris probe designer tool (Femino et al. 1998; Raj and Tyagi 2010).  Probes were designed with 20 bp lengths, a minimum of 2 bp spacing between homology sites, and maximal masking for genomic similarity and problematic RNA sequences.  Probes were labeled with either Quasar 570 or 670 depending on the application.

In the Stellaris hybridization protocol, 5 nmols of oligos were dissolved in 200 µl TE buffer to create a stock concentration of 25 µM probe. KH2 cells were grown on 35mm imaging grade plastic ibiTreat dishes (Ibidi Biosciences). Media were aspirated and washed twice with 1X PBS. After washing, dish was incubated with 2.5 ml of Fixation solution (4% Paraformaldehyde in 1X PBS) at room temperature for 10 min. Cells were washed twice with 1X PBS and incubated in 70% ethanol for 2 h for permeabilization. After permeabilization, 70% ethanol was aspirated and 1 ml wash buffer (2X SSC, 10% deionized formamide) and allowed to stand for 5 min. Freshly made hybridization buffer (2X SSC, 10% deionized formamide, 1 µl/100ul of 25 µm probe and 0.1 g/ml dextran sulfate) were added and incubated for overnight in a humidified chamber at 37 °C. After hybridization, cells were washed twice with wash buffer at 37^o^C for 30 min each. Cells were stained with DAPI used for imaging.

For the HCR Protocol, probe sequences for single molecule RNA FISH for *Hoxa1, Hoxb1*, and *Cyp26a1* mRNA in the HCR protocol were designed by Molecular Instruments and hybridization was conducted, as per manufacturer’s instructions (Choi et al. 2010; Choi et al. 2014). In addition, samples were fixed and permeabilized as discussed for the Stellaris protocol. Samples were pre-hybridized in probe hybridization buffer (50% formamide, 5X SSC, 9mM citric acid (pH 6.0), 0.1% tween20, 50 µg/ml heparin, 1X Denhardt’s solution and 10% dextran sulfate) for 30 min at 65^0^C. 2 nm probes were added to 100 µl hybridization buffer (final concentration 0.2 pmol) and incubated at 45^o^C for overnight in humid chamber. Samples were washed with serial dilution of probe wash buffer 50% formamide, 5X SSC, 9 mM citric acid(pH 6.0), 0.1% tween20, 50 µg/ml heparin) in 5X SSCT (5xSSC, 0.1% tween20, 10% dextran sulfate) at 45^o^C. 500ul of amplification buffer (5X SSC, 0.1% tween20, 10% dextran sulfate and 500 µg/ml salmon sperm DNA) was added and pre-amplified at room temperature. 60 nm of snap cooled hairpins were added to amplification buffer and incubated overnight at room temperature. Excess hairpins were washed with three washes of 5X SSCT. Cells were stained with DAPI.

**Imaging and image analysis for fluorescent *in situ* hybridization**

ES colonies were immobilized on 35 mm imaging grade plastic ibi Treat dishes (Ibidi Biosciences). Images were acquired with a Perkin-Elmer Ultraview spinning disc microscope with a CSU-X1 Yokogawa disc, equipped with a C9100 Hamamatsu Photonics EM-CCD. A 100X 1.4 NA Plan-apochromatic objective was used. The 405 nm, 561 nm and 640 nm laser lines were used to excite DAPI, Quasar 570, and Quasar 670, respectively. Quasar 670 emission was collected through a 455-505 nm, 660-750 nm dual band pass filter, while Quasar 570 and DAPI were both collected with a 415-475 nm, 580-650 nm dual band pass filter. The system was free of cross-talk, as all data was taken in alternating excitation mode, Quasar 570 did not absorb 640 nm excitation, and there was no back bleed- through of Quasar 670 into the 580-650 nm emission filter used for Quasar 570.

For each ES colony, a z -stack was acquired with 0.3 µm steps, spanning 26 total microns. For smaller colonies, this spanned the entire colony. For larger colonies, care was taken to ensure that the acquired region included the region closest to the cover slip.

Data was binned in z by 2, giving a total z spacing of 0.6 µm. Next, a Gaussian blur with radius 0.7 pixels was carried out. A rolling ball background subtraction with a radius of 5 pixels was done in red (Quasar570) and far red (Quasar 670) channels. As the goal was to determine the number of positive cells and relative levels of transcript per cell in the ES colonies, the next step was to segment out cells. Due to the crowded nature of cells in the ES colonies, it was not possible to automate segmentation of DAPI signal in 3D. Therefore, we took a manual approach to segmentation. A max projection was carried out over the 10 slices closest to the coverslip – spanning 6 microns. In most cases, this spanned the bottom layer of cells in the colony. See Supplement Fig. S2 for example images of these projections.

Using DAPI, regions of interest (ROI) for analysis were drawn around each nuclei, including 6 to 10 pixels outside the nucleus to include cytosolic transcripts. On the 6 µm max projection of the red and far red FISH channels, a second rolling background subtraction with a radius of 100 microns was done to remove out of focus florescence and autofluorescence. Puncta from individual transcripts and nascent transcripts were easily visible. To further reduce background from non-specific signal or non-specific probe binding, average intensity was approximated in regions that had no visible puncta, and a value slightly above this was set as a lower threshold. For both the *Cyp26a1*-q570, *Hoxa1*-q670 data set, and the *Hoxb1*-q570, *Cyp26a1*-q670 data set, these values were 2000 and 1500 in the red channel, and far red channel, respectively. At this point, the ROI’s generated from the DAPI channel were applied to the FISH channels, and integrated intensity per cell was recorded.

Box plots were generated for this data for uninduced cells, and cells induced with RA for 4 h or 24 h. For these box plots, the small square is the average, the two lines equally spaced about the average are the standard error of the mean, and the third line in the box is the median. Whiskers are ½ of the standard deviation. For two-color analysis, it was first necessary to determine a cut-off intensity value above which a cell could be considered ‘on’ for expression of a given gene, and below which a cell would be considered ‘off’. For this value, for each gene, we arbitrarily took 90% as the cutoff of intensity from the data of uninduced cells. In other words, for the sake of comparative analysis, any cell expressing more than 90% of the uninduced cells was considered ‘on’. This allowed quadrants to be drawn in the two-color scatter plot, where the white box represents cells off the both genes, the green box represents cells off for *Cyp26a1* but on for *Hoxa1* (top plot) or *Hoxb1* (bottom plot) (Fig. 2), the blue box represents cells on for *Cyp26a1* but off for *Hoxa1* or *Hoxb1*, and the pink box represents cells on for both genes. Note that all plots in Fig. 2 are plotted on log10 scales. Scale bars shown are 10 µm.

Ahn Y, Mullan H, Krumlauf R. 2014. Long-reange regulation by shared retinoic acid response elements modulates dynamic expression of posterior *Hoxb* genes in CNS development. *Dev Biol (San Diego, CA, U S)* **388**: 134-144.

Choi HM, Beck VA, Pierce NA. 2014. Next-generation in situ hybridization chain reaction: higher gain, lower cost, greater durability. *ACS Nano* **8**(5): 4284-4294.

Choi HM, Chang JY, Trinh le A, Padilla JE, Fraser SE, Pierce NA. 2010. Programmable in situ amplification for multiplexed imaging of mRNA expression. *Nat Biotechnol* **28**(11): 1208-1212.

Femino AM, Fay FS, Fogarty K, Singer RH. 1998. Visualization of single RNA transcripts in situ. *Science* **280**(5363): 585-590.

Gautier L, Cope L, Bolstad BM, Irizarry RA. 2004. affy--analysis of Affymetrix GeneChip data at the probe level. *Bioinformatics* **20**(3): 307-315.

Kent WJ, Sugnet CW, Furey TS, Roskin KM, Pringle TH, Zahler AM, Haussler D. 2002. The human genome browser at UCSC. *Genome Res* **12**(6): 996-1006.

Lin C, Garrett AS, De Kumar B, Smith ER, Gogol M, Seidel C, Krumlauf R, Shilatifard A. 2011. Dynamic transcriptional events in embryonic stem cells mediated by the super elongation complex (SEC). *Genes Dev* **25**(14): 1486-1498.

Raj A, Tyagi S. 2010. Detection of individual endogenous RNA transcripts in situ using multiple singly labeled probes. *Methods Enzymol* **472**: 365-386.

R Core Team. 2015. R: A language and environment for statistical computing. *R Foundation for Statistical Computing*, Vienna , Austria.

Rosenbloom KR, Sloan CA, Malladi VS, Dreszer TR, Learned K, Kirkup VM, Wong MC, Maddren M, Fang R, Heitner SG et al. 2013. ENCODE data in the UCSC Genome Browser: year 5 update. *Nucleic Acids Res* **41**(Database issue): D56-63.

Smith KT, Martin-Brown SA, Florens L, Washburn MP, Workman JL. 2010. Deacetylase inhibitors dissociate the histone-targeting ING2 subunit from the Sin3 complex. *Chem Biol* **17**(1): 65-74.

Smyth GK. 2005. limma: Linear Models for Microarray Data. In *Bioinformatics and Computational Biology Solutions Using R and Bioconductor*, (ed. R Gentleman, V Carey, W Huber, R Irizarry, S Dudoit), pp. 397-420. Springer New York.

Thorvaldsdottir H, Robinson JT, Mesirov JP. 2013. Integrative Genomics Viewer (IGV): high-performance genomics data visualization and exploration. *Brief Bioinform* **14**(2): 178-192.
