# Supplementary material for: Analysis of dynamic changes in retinoid-induced transcription and epigenetic profiles of murine Hox clusters in ES cells
Source: Genome Res. 2015 Aug;25(8):1229–43. doi: 10.1101/gr.184978.114 (PMC4510006; doi:10.1101/gr.184978.114)
Supplement: Supplemental Material [file supp_gr.184978.114_Table_S6.docx]

| **Assay name** | **Isoform** | **F primer** | **R primer** | **Genomic coordinate** |
| --- | --- | --- | --- | --- |
| [GAPDH](http://www.ensembl.org/Mus_musculus/exonview?db=vega;transcript=OTTMUST00000067084) |  | TGGCCAAGGTCATCCATGA | CAGTCTTCTGGGTGGCAGTGA | NM_008084 |
| [ATP5B](http://www.ensembl.org/Mus_musculus/exonview?db=core;transcript=ENSMUST00000026459) |  | TTGACAACATCTTCCGCTTTACC | AAGGGATTCTGCCCAATAAGG | NM_016774 |
| Halr1  (161) | 1 | AAGCCAAGCTCAGGTGTGTT | TGTGTGATCTGGGAAGCAAG | chr6:52063401-52063561 |
| Halr1  (115) | 2 | ACCCGAGTTTACCAGGTTCC | TCAAAGACCCAGGTGAGAGG | chr6:52062372-52062486 |
| Halr1  (125) | 3 | CTGTAGCCTCACCACACAGG | GGAACCTTTTCTGAGGTTCACT | chr6:52061956-52062080 |
| Halr1  (110) | 4 | TAAAGGCTCAGCAGGGGTTA | TGGAACCTTTCTTGGTGAGC | chr6:52056369-52056478 |
| Halr1  (158) | 5 | CATGATGGTGATGTCGAAGG | CTGAGCTGGACGGAGAACA | chr6:52058270-52058427 |
| Halr1os1 (60) | 1 | TCAGAACTGTATTCTTAGGGAGCA | GGGGTGGGAAGTTCACTG | chr6:52055667-52055726 |
| Halr1os1 (102) | 2 | TTTTCACAAGGCTTGATGTGTC | GAGTGAAGTCACAAATGTCACCA | chr6:52056782-52056883 |
| Hotairm1 (165) | 1&2 | CCTTCTCACGCTTCTTCTGC | ATTTCACCACCAAGCAGCTC | chr6:52106873-52107037 |
| HotAirm2 (200) |  | CCGATGTGGATGAAGGAGTT | GCTACTCCAGCCCAACTCTG | chr6:52106532-52106731 |
| Hobbit1  (196) |  | TTGACTGAGGCCTGCTTTCT | CGCCTTTGCTTTGATTTTTC | chr11:96175506-96175701 |
